# Toward using cosmic rays to image cultural heritage objects

**DOI:** 10.1016/j.isci.2025.112094

**Published:** 2025-02-25

**Authors:** Andrea Giammanco, Marwa Al Moussawi, Matthieu Boone, Tim De Kock, Judy De Roy, Sam Huysmans, Vishal Kumar, Maxime Lagrangev, Michael Tytgat

**Affiliations:** 1 Centre for Cosmology, Particle Physics and Phenomenology (CP3), Université catholique de Louvain, Chemin du Cyclotron 2, 1348 Louvain la Neuve, Belgium; 2 Ghent University Centre for X-ray Tomography, University of Ghent, Proeftuinstraat 86, 9000 Gent, Belgium; 3 Antwerp Cultural Heritage Sciences (ARCHES), University of Antwerp, Mutsaardstraat 31, 2000 Antwerp, Belgium; 4 Stone Sculpture Studio, Conservation & Restoration, Royal Institute for Cultural Heritage (KIK-IRPA), Jubelpark 1, 1000 Brussels, Belgium; 5 Elementary Particle Physics Group, Vrije Universiteit Brussel, Pleinlaan 2, 1050 Brussel, Belgium

**Keywords:** Particle physics, Elementary particles, Particle astrophysics

## Abstract

Muons are elementary particles abundantly and freely produced in cosmic-ray interactions and can be used for nondestructive imaging of internal structures. This novel technique, nicknamed “muography”, has been applied in use cases ranging from geophysics to archaeology to nuclear safety, but it has been so far underexplored for a vast category of cultural heritage objects that are relatively dense (stone, metals, etc.) and with size ranging from decimeters to human size. In this paper, we review recent development of portable muon detectors and explore the strengths and limitations of the technique. Despite the limited particle flux available, muography is found to be an intriguing option, particularly in cases where the items to be analyzed are not transportable or set up in a confined environment.

## Highlights

• Muography is a harmless imaging technique based on cosmic radiation
• We propose portable muography as a tool complementary to standard techniques
• We review the state of the art and explore strengths and limitations
• It is a promising option for cultural heritage objects that are not transportable

## Graphical abstract

**Figure:**
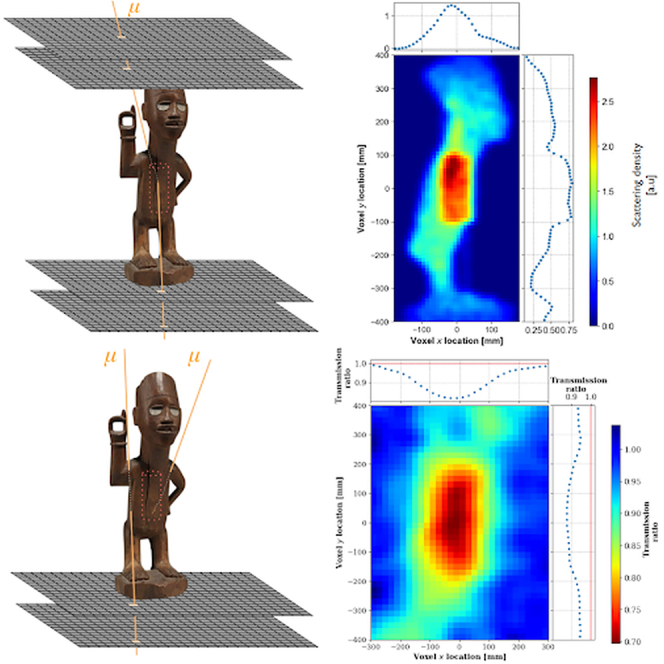

## Introduction

In the past decades, imaging techniques based on elementary particles have been extensively used in the investigation and analysis of cultural heritage. The most ubiquitous examples are based on X-rays^1^: imaging sculptures/objects to visualize their composition (different volumes, joints, old restorations, inner structures, or reinforcements); imaging mummies, reliquaries, tombs to visualize their content without manipulating the objects; dating wooden artifacts using dendrochronology; etc. However, there are important limitations to X-ray imaging. When looking at large objects, or objects composed of dense materials (compact stone, metal, etc.), the penetrating power of standard X-rays is insufficient.

By using alternative types of radiation, such as MeV-range X-rays and neutrons,^2^^,^^3^^,^^4^ this problem can be partly overcome. However, moving valuable objects to an imaging facility is not always feasible due to their size, weight, nature, poor state of preservation, or simply the risk of damage during transport. These limitations affect both movable and immovable heritage such as monuments, monumental statues and building decorations, and individual sculptured objects. Nevertheless, imaging is key for a better understanding of their composition and can provide important information about their construction techniques, earlier interventions (gluing, reinforcements, additions, etc.), their condition and interrelationships with the environment.^5^ Portable and compact setups for X-ray fluorescence analysis (XRF) are available for use in cultural heritage studies,^6^^,^^7^ where fluorescent X-rays are used to reveal chemical composition, but are intrinsically limited to shallow depths by the high absorption rate of X-rays. Portable X-ray computed tomography (CT) systems have been deployed for the *in situ* study of art objects.^8^ However, these are less performant than fixed laboratory systems and require safety measures during the data-taking campaign. Neutron absorption reaches larger depths than X-rays,^9^ and portable fast-neutron sources are also in use.^10^ A portable proton accelerator has been recently developed^11^ for *in situ* X-rays (PIXE, Particle Induced X-rays Emission) and low-energy *γ*-rays (PIGE, Particle Induced Gamma-rays Emission) detection. All these methods suffer the practical issue that ionizing radiation (including X-rays, *γ*-rays, protons, and neutrons) is strictly regulated because of radiation hazard concerns, and neutrons can also cause the activation of material (i.e., creating radioactive nuclei inside the object under investigation), which is highly undesirable.

These shortcomings have led to the investigation of imaging methods based on a different elementary particle, the muon (*μ*). What makes muons unique is their very high penetration power in matter: differently from neutrons and protons they are insensitive to the strong nuclear force, and being about 200 times heavier than electrons they lose much less energy by electromagnetic processes such as ionization and bremsstrahlung. Only neutrinos penetrate more than muons in matter; but while neutrinos are very hard to detect, muons are actually easy to identify and track by modern particle detectors.

A few facilities in the world, e.g., CERN and PSI in Switzerland, ISIS in the UK, TRIUMF in Canada, FNAL in USA, and J-PARC in Japan, are able to produce muon beams with precisely controlled energy and high intensity. Artificial muon production typically starts with the collision of energetic protons with the protons and neutrons bound in nuclei to produce charged pions and kaons, which in turn decay into muons.^12^ While mostly produced for the purposes of fundamental physics experiments, these muon beams can be used for applications in other fields, including elemental analysis for cultural heritage studies. A technique called Muonic Atom X-ray Emission Spectroscopy (*μ*-XES) or Muon Induced X-ray Emission (MIXE) exploits beams of negative muons at sufficiently low energy to be stopped (through energy loss by ionization) inside the object of interest. Stopped negative muons form so-called “muonic atoms” by displacing an electron in one of the outer atomic shells, and these atomic systems relax to their lowest-energy state by emitting X-rays, which are in turn detected and whose spectra depend on the atomic number *Z* of the material.^13^^,^^14^^,^^15^^,^^16^ With respect to the conceptually similar XRF, PIXE, and PIGE techniques, MIXE has the advantage of probing deeper in the material, thanks to the large muon penetrating power. Moreover, activation of the material is negligible with respect to irradiation with neutrons or protons. While this method is very promising for the analysis of relatively small objects, it suffers a fundamental limitation: The objects to be analyzed have to be transported to one of the few existing muon beam facilities in the world. Some very ambitious ideas have been proposed toward a transportable muon generator,^17^ but artificial muon production must necessarily start from collisions and decays of other particles, which implies radiological hazards, like all methods based on an artificial particle source, because of the byproducts of the collisions.

In this paper, instead, we argue for portable and safe imaging based on naturally available muons of cosmic origin, to address use cases where other methods are insufficient or inadequate or simply not usable because of practical constraints (e.g., in transportability). Mostly produced at high energy (most of them at order GeV) by the interaction of cosmic rays in our planet’s atmosphere, cosmogenic muons easily penetrate through large depths^18^; they are harmless and available everywhere. These characteristics inspired several practical applications in sub-surface imaging, collectively known under the umbrella term of “muography”^19^^,^^20^^,^^21^^,^^22^^,^^23^^,^^24^ (or cosmic-ray muography, to distinguish it from the accelerator-based muon imaging technique outlined in the previous paragraph). Two main muography techniques exist, to which in this paper we refer as absorption-based and scattering-based.

**Figure 1:**
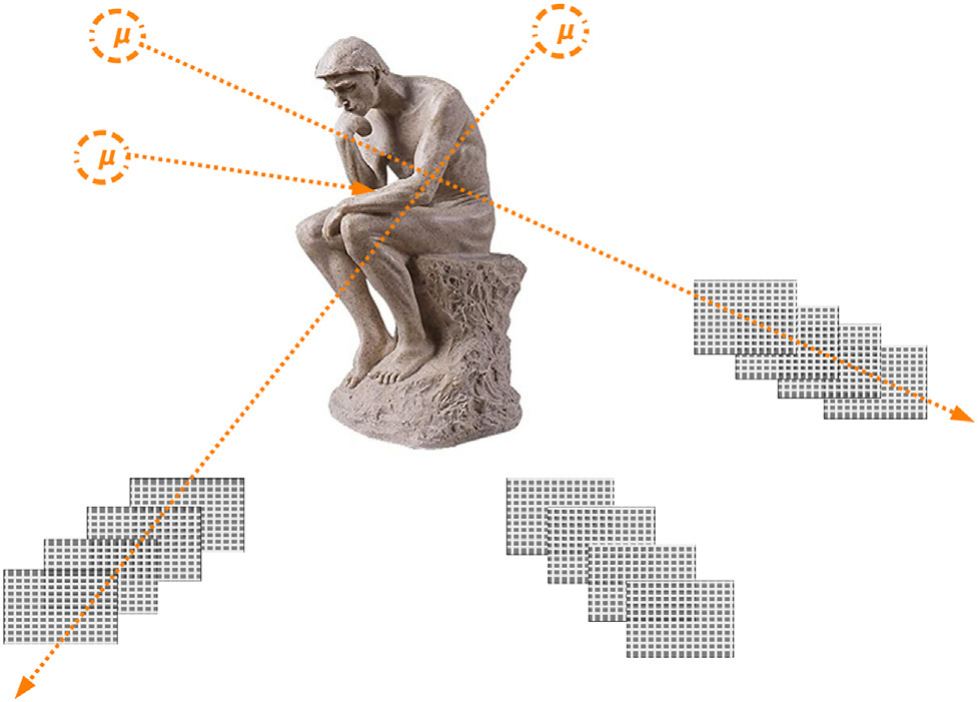
In absorption muography, muon trackers are downstream of the object of interest; 3D imaging can be obtained by combining multiple viewpoints Reproduced from.^25^

Absorption(-based) muography^24^ exploits the loss of energy (mostly by ionization) of muons when passing through any material; when its initial kinetic energy is completely exhausted, the muon stops and decays into an electron and two neutrinos, which are not detected.^18^ (For sake of brevity, and following the conventions of high energy physics, through this paper we employ the word “electron” to also indicate positrons, i.e. anti-electrons, and with the word “muon” we do not make distinction between negatively and positively charged muons, although the latter are also called anti-muons). Absorption muography is directly sensitive to the matter density of the material, which gives the possibility to discriminate between different hypotheses on the inner composition of a large object, and also assess specific conditions such as the moisture content, as we elaborate in this paper. Similar to conventional radiography, absorption muography naturally yields 2D images. In this technique, the trajectory of the muon must be recorded by a tracker, which measures its zenith (*θ*) and azimuth (*ϕ*) angles. The tracker is usually an array of position-sensitive layers of particle detectors, although other tracker types exist.^20^ Subsequently, the 2D distribution of the muon rate in the θ−ϕ plane is compared between datasets with and without the object of interest in front of the tracker, to get a transmission map, which in turn can be translated into density projections or opacity maps (where the opacity is defined as the integral of density over a line of sight). To obtain 3D images, 2D density projections observed from different points of view can be combined through inversion algorithms. A possible arrangement of muon trackers around a statue, for 3D imaging, is sketched in Figure 1.

**Figure 2:**
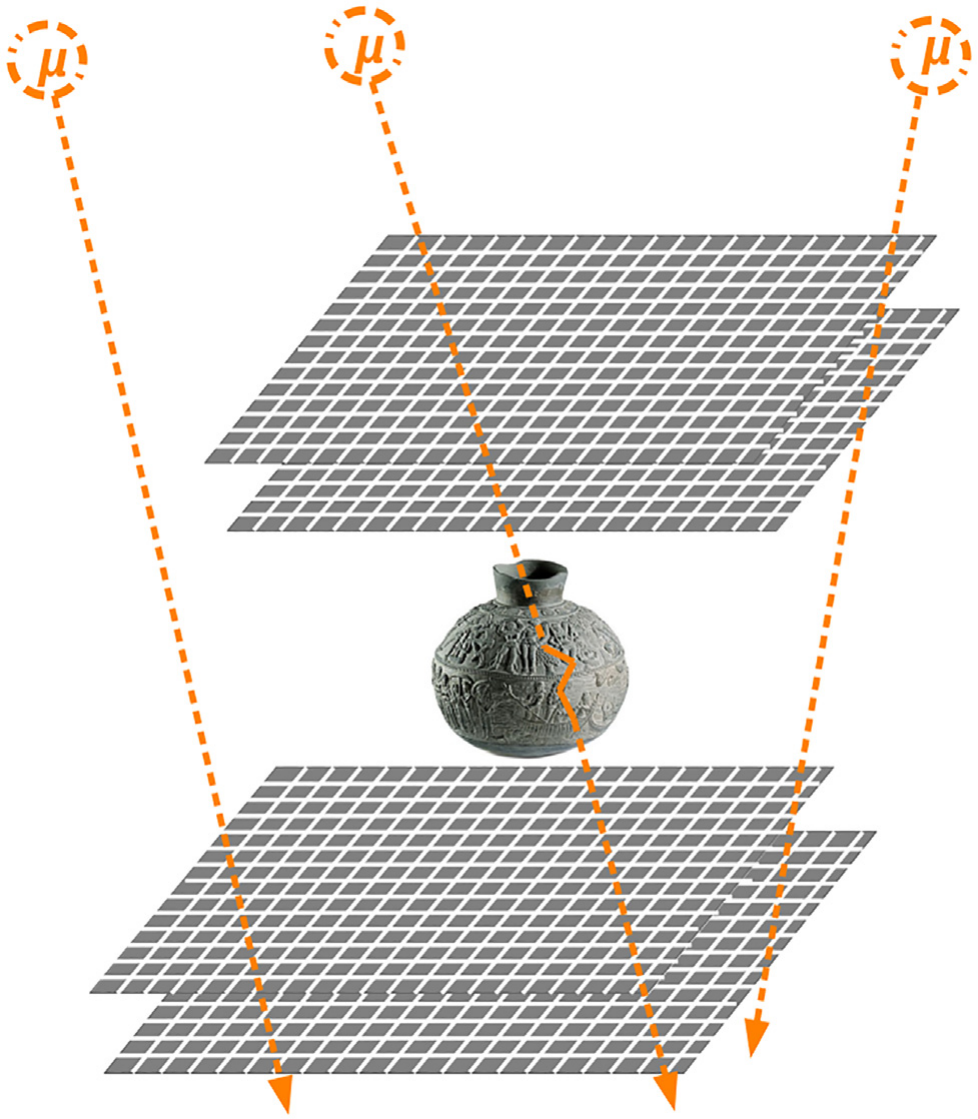
In scattering muography, the object under investigation is “sandwiched” between muon trackers Reproduced from.^25^

**Figure 3:**
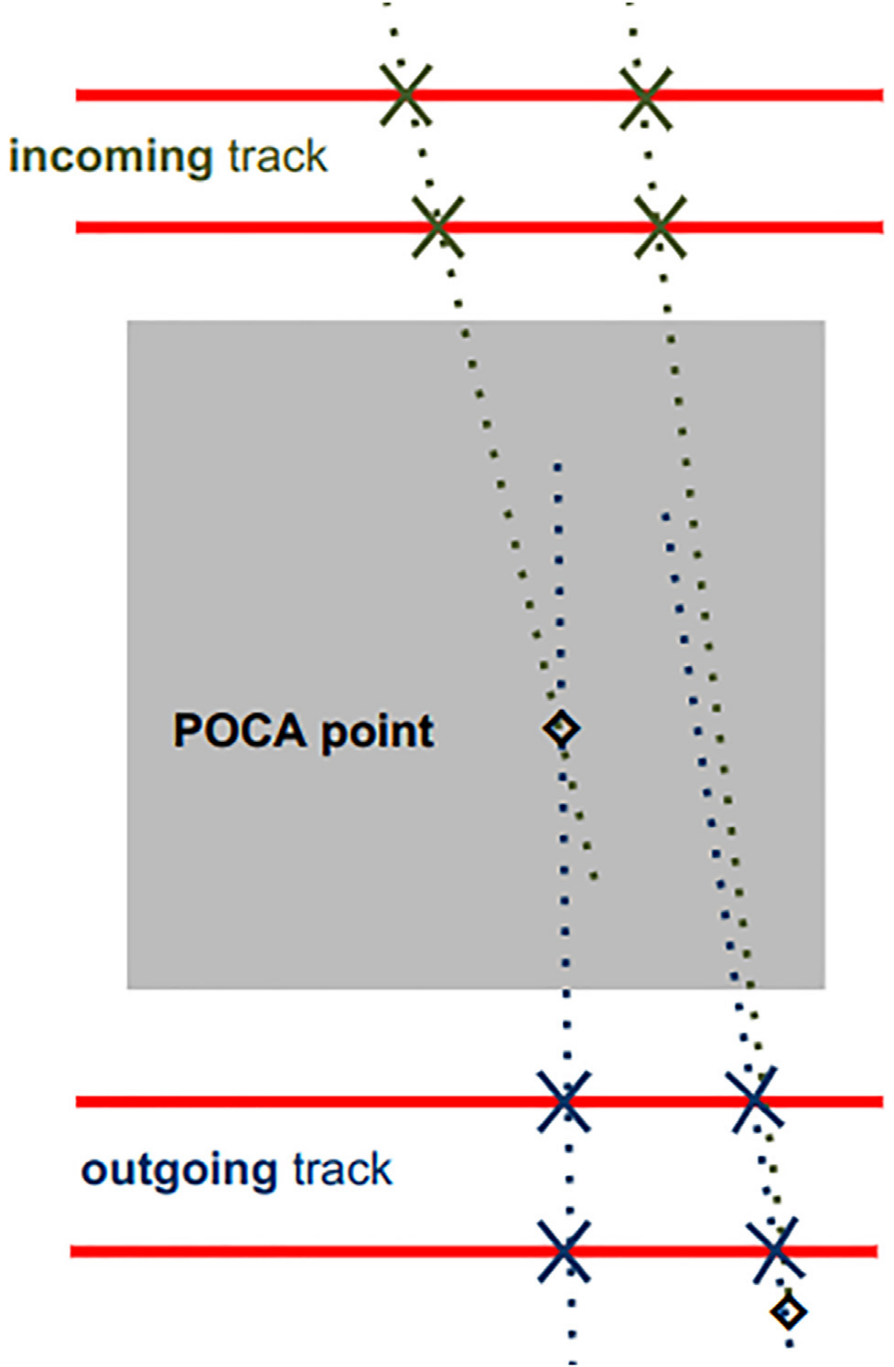
Sketch of Point of Closest Approach (POCA) calculation Left: POCA reconstructed inside the volume of interest. Right: POCA reconstructed outside the volume of interest.

Scattering(-based) muography, first proposed in 2003,^26^ instead exploits the diffusion of muons due to the electric charge of the atomic nuclei in matter. The average angular deviation of the muon trajectory has a very strong dependence on the atomic number *Z* of the substance traversed,^18^ i.e., the number of protons in the nucleus of its atoms, which means that this method can be used for elemental discrimination. In practice, the muon trajectory must be reconstructed both upstream and downstream of the volume of interest in order to measure the angular deviation. As a consequence, the object under investigation must be “sandwiched” between muon trackers, as illustrated in Figure 2, which becomes impractical or expensive when the object is large. Scattering muography is an intrinsically 3D imaging method, as it is possible to reconstruct the muon trajectory in 3D space. In the most popular analysis method for scattering muography,^19^ the most likely position of the largest scattering event is estimated for each muon by extrapolating the incoming and outgoing trajectories measured by the two trackers and finding their point of closest approach (POCA),^27^ as illustrated in Figure 3. From the frequency of POCA points attributed to each voxel, and the average scattering angle in each, it is possible to test hypotheses on the identity of the material voxel by voxel. This POCA method implicitly assumes that only one large-scattering event happened and that all other scattering events can be neglected; although this approximation is satisfactory for relatively small objects, several other algorithms have been developed in the specific literature that do not rely on this assumption.^28^

Each of these two muography techniques has its own sensitivity, applicability, and limits. In absorption muography, a single muon tracker is able to measure the 2D projection of matter density, and the combination of measurements from different viewpoints can give a 3D density map. However, it provides no material discrimination apart from density, and small-size or low-density objects do not stop enough muons to provide sufficient contrast. In scattering muography, at least two muon trackers are needed, upstream and downstream of the object of interest, to reconstruct the muon trajectory before and after passing through it. This method naturally yields 3D information, and is sensitive to elemental composition because the width of the scattering angle distribution is a function of atomic number *Z*. However, it is impractical for human-sized statues, as the object of interest must fit between the two trackers. Either the object of interest is moved inside the set-up, or a rather complex installation of the detectors must be performed around the object. Therefore, this method is appropriate for relatively small objects.

Cosmic muons pose no hazard, being part of the natural and ubiquitous background that constantly crosses our bodies and the objects of art. Muon detectors can be made relatively inexpensive and transportable. Muography images can be interpreted in terms of a few parameters such as density and *Z*. Generally speaking, 3D inversion of muography data suffers from less ambiguities and degeneracies, hence requires less boundary conditions to achieve convergence, than some other remote sensing techniques. But muography also has some fundamental limitations: the muon flux is relatively small, of the order of 100 ${\text{Hz}/m\;}^{2}$; the scattering in matter blurs the images, and the amount of blurring depends on the muon energy, whose spectrum is very model-dependent and which is hard to measure per particle without making the size and cost of the detectors increase drastically. For these reasons, it may be unrealistic for muography to supersede well-established remote sensing methods (which already reached technological maturity) in the use cases where they have been applied since long time; instead, our aim is to identify and possibly fill some gap in usability of the other methods.

This paper aims at serving as a review while at the same time proposing new possible use cases for muography, supporting them with a simulation study that explores the ranges of applicability of this technique. The rest of this document is structured as follows: state of the art in portable non-destructive test methods for cultural heritage summarizes the non-destructive quantitative analysis methods used in cultural heritage preservation that fulfill the request for portability, while state of the art and developments of interest in muography presents examples of prior applications of muography to (large-scale) cultural heritage. In results, we present a simulation study (expanding the preliminary one presented in ^25^) whose aim is to identify in which regimes the two main muography methods are most promising, and which limitations must be overcome in future studies. We discuss the path ahead in limitations of the study, in particular, what technical and methodological developments are needed in order to make muography a reliable technique for the applications discussed in this paper. Proposal for future case studies elaborates on a few future case studies of great interest for cultural heritage preservation. In conclusions, we summarize the key findings of this article. Finally, STAR Methods provides additional explanations on the algorithms employed in our simulation study.

A set of appendices completes this paper. The interested reader will find the essential formulas for absorption- and scattering-based muography in basics of muography. Momentum measurement explains how muon momentum can be roughly measured to improve muography images. Statistical considerations for low-size objects presents some statistical considerations about low-size objects that have not been discussed in the literature to the best of our known knowledge.

### State of the art in portable non-destructive test methods for cultural heritage

A condition assessment is indispensable for the conservation of cultural heritage objects. Whether it is a piece of art, a monument, or another object with cultural value, this starts with a deep visual observation of the object, supported by a detailed photographic recording of the object to document the state of preservation at that time. Surface 3D-models can be achieved easily, apart from 3D scanning techniques, by combining different views from different angles from the object, a technique known as photogrammetry^29^ (whose application to muography has been reported, in an archaeological context, in^30^). These 2D or 3D images serve as the base for detailed mappings of observed materials, surface treatments and pathology. Additional portable methods are available for more profound diagnostics.

#### Metal detector

Non-ferrous and ferrous metal elements, like inner supporting structures in sculptures, can be detected by a portable metal detector (stud or wall detector), designed for the detection of wires, cables and pipes in buildings. It is a handheld device that sends an electric signal that interacts with the density and composition of an object in a building material, a sculpture or an architectural decoration. These electronic detectors use sensors to detect any changes in the dielectric constant of the support. The dielectric constant will change when the scanner sensor passes over the stud. This device is often limited to a maximal depth of 12–15 cm for ferrous metals and even less for non-ferrous metals. It is possible to locate the center of the metal element but no information can be obtained about the dimensions and the exact orientation of the metal elements. In the case of sculptures, the use of the detector can be obstructed by the complex form or composition of the sculpture itself.

#### Radar

Imaging radar technologies, in particular ground penetrating radar (GPR) detect subsurface dielectric discontinuities through the reflection of emitted electromagnetic waves. Originally developed for geophysical surveying, applications in cultural heritage are mainly oriented toward structural investigations.^31^^,^^32^

#### IR-thermography

Infrared thermography measures the infrared (thermal) radiation coming from the material under investigation and renders the image of the surface area in colors or in gray scale, in relation to a temperature scale for an assumed material emissivity.^33^ It can be used to spot surface anomalies, but it is often difficult to interpret observations without the support of optical imaging or other techniques. It has been used to detect near-surface differences in moisture conditions, as evaporation of moisture chills the surface relative to drier areas.

*Ultrasonic pulse velocity (UPV)* measurement is a technique that measures the travel time of an ultrasonic pulse (typically a P-wave), sent by a transmitter and captured at a known distance from the transmission source. The UPV is the ratio between the path length (the distance between transmitter and receiver) and travel time. Anomalies such as fissures, cracks, cavities, and decohesion reduce the UPV relative to sound material, allowing for their indirect assessment.^34^ Some standard materials, like Carrara marble, have well characterized UPV values supported by multiple studies, while others need benchmarking case by case, putting limits on its usability. The distance between the transmitter and receiver is dependent on the wave frequency, but typically requires >10 cm. In case of a severely damaged sculpture (for example the Egyptian Khonsou sculpture, see proposal for future case studies later in this article) it is impossible to interpret these values in order to determine the pattern of the cracks. Some authors have argued other parameters, such as spatial attenuation, to be more reliable.^35^ However, that would require the measurement setup to be equipped with a (more expensive) oscilloscope, which is not always the case.

*Ultrasonic tomography* is based on a series of UPV measurements. With this technique it is possible to define areas in objects where fractures and cracks are present. Ultrasonic tomography is mostly used in concrete industries but some studies have been performed on cultural heritage.^36^^,^^37^ This technique is often used on two-dimensional views at a certain depth of the object but also three-dimensional analyses are possible. 3D scanning of the surface increases the potential use of ultrasonic tomography, as this can serve as a base to determine the location of transmitter and receiver as it simplifies the identification of the x, y, and z positions of the transmitter and receiver of the ultrasonic waves on the statue. Nevertheless, these measurements are labor intensive and become more complex with increasing complexity of the object’s shape. As measurements must be taken on the surface of the object under investigation, this must be fully accessible along all sides.

### State of the art and developments of interest in muography

The first application of muography dates back to the middle of the past century,^38^ to measure the overburden of a tunnel. It then took 15 years to see the second, and arguably most famous, application of the same principle, when L. Alvarez’s team used a spark chamber as a muon tracker to search for possible hidden chambers inside an Egyptian pyramid.^39^ More applications followed, especially in the 21st century, and muography is nowadays a booming research area, with a steadily growing trend of publications.^40^ The interested reader can refer e.g., to the reviews in ^20^ and,^21^ two recent books^22^^,^^23^ and a primer.^24^ Several ongoing efforts in absorption-based muography focus on targets that are very large, i.e., from tens of meters (e.g., buildings - both ancient and modern - and civil infrastructure such as bridges) to kilometers (mountains, and in particular volcanoes),^22^ while after the invention of scattering-based muography in 2003,^26^ the latter technique has been applied or advocated for many uses in the nuclear safety sector (e.g., nuclear waste assays, nuclear smuggling prevention at border controls, verification of non-proliferation treaties), exploiting its unique sensitivity to the number of protons in nuclei. Because of the need to “sandwich” the volume of interest between upstream and downstream detectors, scattering muography is generally limited to smaller objects than absorption muography.

Muography has already been successfully used for studies of large-scale cultural heritage. One of the most renowned examples is the ScanPyramids project, based on three different detector technologies (plastic scintillator detectors, micro-pattern gaseous detectors, nuclear emulsions), which in 2017 announced an unexpected low-density anomaly in Khufu’s Great Pyramid.^41^ Additional data have been collected since then, leading to the recent announcement of a corridor-like structure of about 9 m length with a transverse section of about 2 m by 2 m.^42^ Confidence in the solidity of these conclusions was given by the addition, since 2020, of measurements with GPR and ultrasonic testing.^43^ This motivated finally the visual inspection via an endoscope, which gave uncontroversial confirmation of the claim.

In another example, density anomalies (potentially posing safety hazards) have been found in a rampart of a defensive wall of Xi’an (China).^44^ In the Svyato-Troitsky Danilov monastery (Russia), areas of significantly higher density within immured vaults (interpreted as walls and partition walls) and unknown voids (possible ancient crypts or air ducts) have been revealed through the use of muon detectors based on nuclear emulsions.^45^ Nuclear emulsions have also been exploited in a muography survey of an archaeological site located in a highly populated district of Naples (Italy), ten meters below the current street level, which observed some unknown structures, including one compatible with the hypothesis of a hidden, currently inaccessible, burial chamber.^46^

All the above examples made use of absorption muography, and in general many methodological developments from geophysical applications (see ^22^^,^^47^ for dedicated reviews) can also be applied to the study of very large human-made objects. However, a proposal has been made to search for iron chains within masonry in the dome of the Florence cathedral (Italy)^48^ using scattering-based muography, and a proof-of-principle test has been carried out on a mock-up wall, demonstrating the conceptual validity of the method. Another method has been invented more recently, alignment-based muography, which exploits the fact that, in the absence of electric or magnetic fields, the muon trajectories are straight lines.^49^ This method requires two muon telescopes, positioned respectively on a fixed reference system (e.g., a structural element of a building) and on the point whose movements need to be monitored. If the actual position of the latter is not where the track reconstruction algorithm expects it to be, the muon’s half-trajectories do not meet, and the degree of misalignment can be monitored as a function of time as part of an alarm system for the stability of the building. A proposal has been made to employ this method for the long-term monitoring of the stability of Palazzo della Loggia in Brescia (Italy).^50^

In all these examples, the particle detectors are relatively large, and therefore not easily transportable. This is due to the large size of the objects of interest, which implies a large attenuation of the flux of muons passing through it, which demands a large cross-sectional area (order $m^{2}$) in order to maximize the usable statistics within a reasonable data-collection time. Instead, in the use cases outlined in discussion, we argue for the development and deployment of compact and portable particle detectors. Given the confined space available in e.g., a museum or a church, they must be small. Additionally, researchers in some cases may be granted access only outside of visiting hours, therefore, the detectors must easily deployable and removable. And when we need to resolve relatively small features within the object, as in the examples of bulk features in monuments and artwork and inner cracks, spatial resolution of the detectors is an important factor. It is also to be noted that the analysis of low-size objects via muography requires a statistical treatment that is different from the more common large-sized ones, as we elaborate in statistical considerations for low-size objects.

A few teams are developing portable muography detectors. For usage underground (e.g., mining exploration), very specialized detectors are developed that fit inside boreholes (first proposed in^51^; see e.g.,^52^ for a recent example); we do not elaborate on those, as the case studies outlined in proposal for future case studies are not underground. R&D projects of portable detectors that would be in principle suitable for the applications that we propose include, among others: MIMA,^53^^,^^54^^,^^55^^,^^56^ based on scintillating bars coupled with silicon photomultipliers; MUST2,^57^^,^^58^^,^^59^ a time-projection chamber (TPC) based on micropattern gaseous detectors called MicroMegas; a project at the University of Kyushu^60^^,^^61^ based on scintillating fibers; a project at Wigner RCP^62^ based on multi-wire gaseous detectors; and our own detector project based on resistive plate chambers, whose various development steps have been detailed in a few proceedings.^63^^,^^64^^,^^65^

## Results

This paper advocates for the adoption of portable and safe muography as a promising imaging approach for cultural heritage studies in a regime that is new for muography (relatively low size) while being beyond reach for methods based on other radiation sources. We aim at demonstrating that both scattering and absorption muography techniques can play a role in cultural heritage imaging as they are complementary in terms of detection apparatus cost, imaging capabilities, and target object size. To illustrate the potential applications and limitations of muography, we report in this section a preliminary imaging study based on simulated muon tomography data. This study updates and expands the preliminary results presented in.^25^

All data used in this study have been generated using the Cosmic-ray Shower Library (CRY^66^) and GEANT4^67^ software programs. CRY is a parametric simulation, specialized in atmospheric cosmic-ray particles, which is popular in muography studies as it generates muons with a good trade-off between accuracy and speed.^68^ The muon flux, especially at low momentum, is influenced by geomagnetic effects that depend on location and time.^18^^,^^20^ CRY allows the user to define altitude, latitude and date as input parameters for the flux calculation.^66^ A detailed simulation of the muon flux is not crucial for the study presented in this document, but it may be of relevance for actual measurements in the future, especially if performed with large data statistics. In this study, we simulate the cosmic muon flux at the sea level, at a latitude of $48^{{}^{\circ}}$ (corresponding to Belgium), and at a random date in year 2012. GEANT4 is a very popular software in particle and nuclear physics, for the detailed simulation of the passage of particles through matter. In this study, GEANT4 is used to simulate the passage through both the object of interest and the sensitive elements of the muon detectors of the muons generated by CRY. The simulated detector planes have an efficiency of 98 % and a spatial resolution of 1mm, which is quite typical of gaseous tracking detectors employed in particle physics.^18^^,^^69^ The trackers have an angular resolution of 2.6 mrad. The amount of simulated muons is 5 millions, which roughly corresponds to 8 h of continuous data taking.

The following subsections show the imaging performance of scattering and absorption muography using various image reconstruction, clustering and image processing algorithms. Three scenarios are investigated: in two of them the object of investigation is a small stone statue with a metallic insertion, and the two scenarios only differ by a factor two in scale, while in the last scenario we simulate a thick wall with a sub-volume having a different moisture content than the rest.

**Figure 4:**
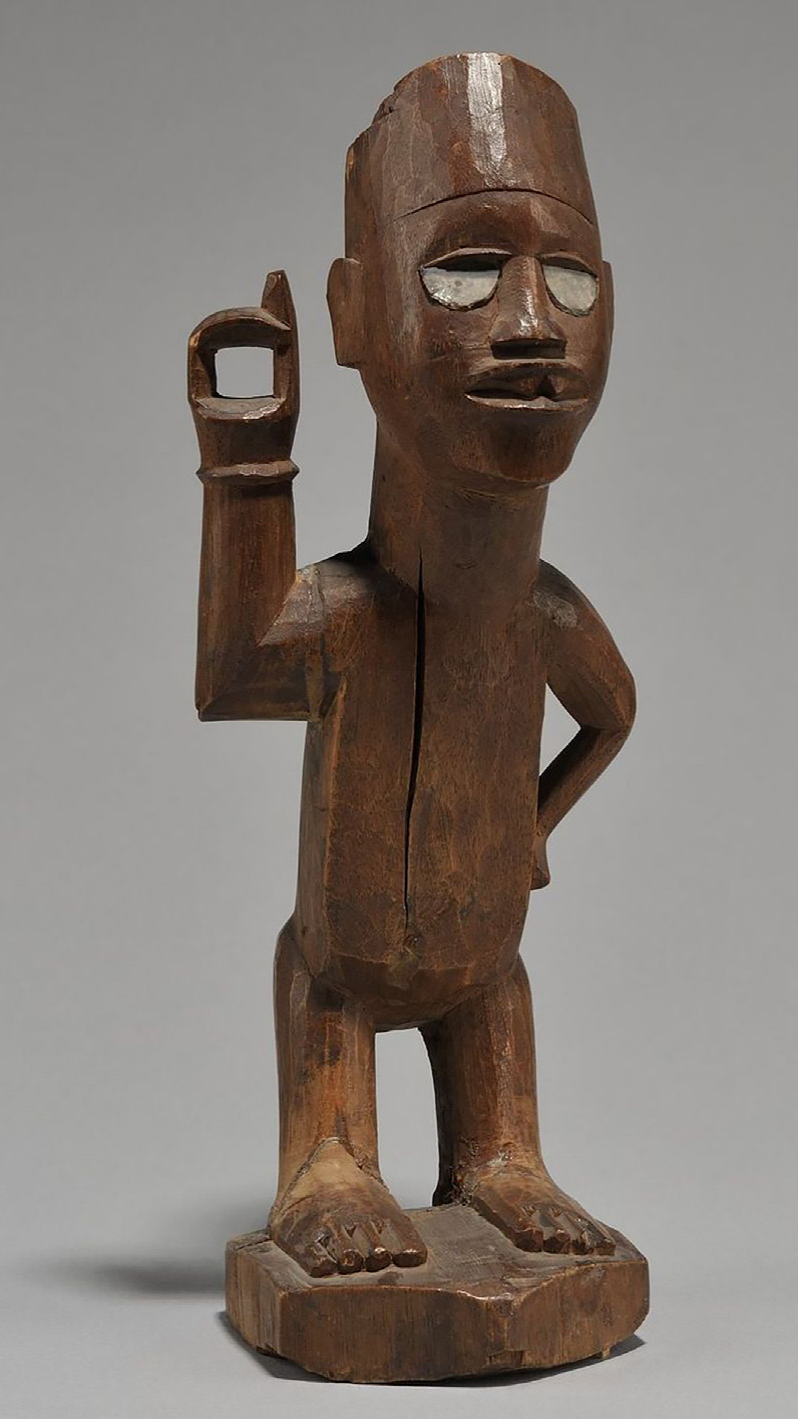
Picture of the wooden statue at the Africa Museum of Tervuren that we used as model From project ToCoWo (https://tocowo.ugent.be/).

In order to have a complex and realistic shape to use as representative of a typical statue, the geometrical model of the African statue shown in Figure 4 was implemented in GEANT4 as a mesh file obtained from a high-resolution CT scan (voxel size 121 *μ*m) performed at the HECTOR scanner of the Ghent University Center for X-ray Tomography.^70^ Although the actual statue is uniformly made of wood, we produced a variety of simulated datasets, some of them where the material is wood (with various moisture levels) and others where the material was changed to marble with a density of $2.71g/{\text{cm}}^{3}$ (The exact composition that we simulated consists of 40% CaO, 25% $SiO_{2}$, 30% $MgCO_{3}$, and 5% $Al_{2}O_{3}$, which is representative of typical marble compositions, see e.g.,^71^). Marble is chosen as main reference material in this study, because it is dense enough that even with small size we can discern the inner features, and on the other hand we can expand the size and still be realistic, because it is common to have large-size statues made of marble in our cultural heritage. Wood, on the other hand, is very light and is thus challenging for muography, and we used it to explore the limits of the method. To investigate the potential of muography for material identification, which may be relevant e.g., to identify the inner tube system of a monumental fountain, we simulate the insertion of hidden cylinders of bronze within its internal structure. The first two scenarios discussed in the next subsections (2.1 and 2.2) correspond to two size regimes, where the statue keeps its actual size and is scaled by a factor two, while the radius of the bronze cylinder is respectively 5 and 10 cm. Details on the third scenario are in the corresponding sub-subsection (2.3). In all three scenarios, we also used Blender-to-Geant4 (B2G4),^72^ a software that facilitates the simulation of complex shapes in GEANT4 by interfacing with Blender^73^ (a popular open-source software for 3D modeling and rendering).

**Figure 5:**
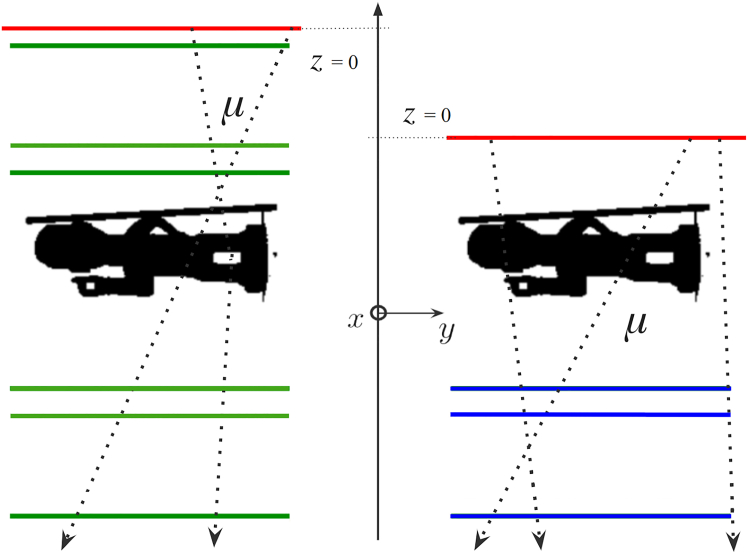
Geant4 simulation setup for the first two scenarios; green and blue panels represent the scattering and absorption setups, respectively The horizontal surface from which the muons are generated is represented by the red line.

In this first exploratory study, we model a detection apparatus made up of six or three planes, for scattering or absorption muography respectively, as illustrated in Figure 5.

### Scenario I: small size regime

The statue’s size simulated in this scenario is $80\times 30\times 30\;{\text{cm}}^{3}$, which is quite small by muography standards, making the scattering method the most relevant. This section presents the imaging capabilities of various scattering tomography algorithms complemented by algorithms relevant for material identification.

As described in introduction, scattering muography is based on the measurement of muon deflections through the object. The scattering angle is measured by extrapolating the incoming and outgoing trajectories measured by the two sets of detectors and used by the reconstruction algorithms to infer the relative density of the target volume. While a plethora of algorithms are available in the literature, this first section will rely on the Point Of Closest Approach (POCA),^74^ which is detailed in STAR Methods.

**Figure 6:**
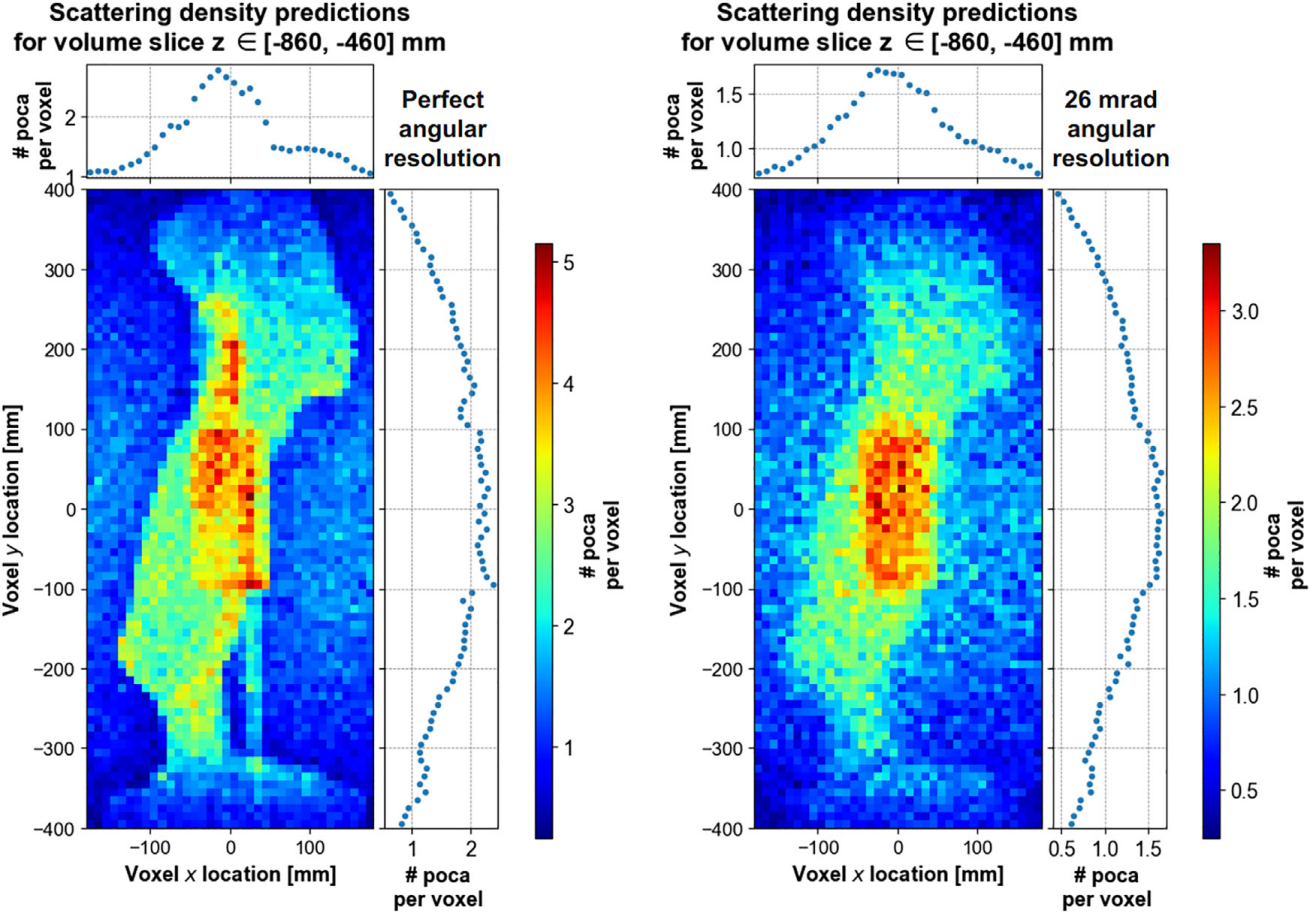
Inferred scattering density in Scenario I, computed as the mean number of POCA points per voxel across the *z* direction for a perfect angular resolution (left) and 2.6 mrad angular resolution apparatus (right), for a 10mm voxel size

The volume at study is divided into three-dimensional pixels (voxels) and the number of POCA points per voxel is used as density prediction. Figure 6 shows a comparison of scattering density predictions obtained with a perfect angular resolution apparatus and the 2.6 mrad angular resolution apparatus. While the reconstruction of the POCA points location isn’t perfect even with an ideal detector (a problem that is mitigated by some smarter algorithms, reviewed e.g., in^28^), the fundamental limitation comes from the angular resolution of the apparatus, which results in many non-empty voxels in the empty space around the statue.

**Figure 7:**
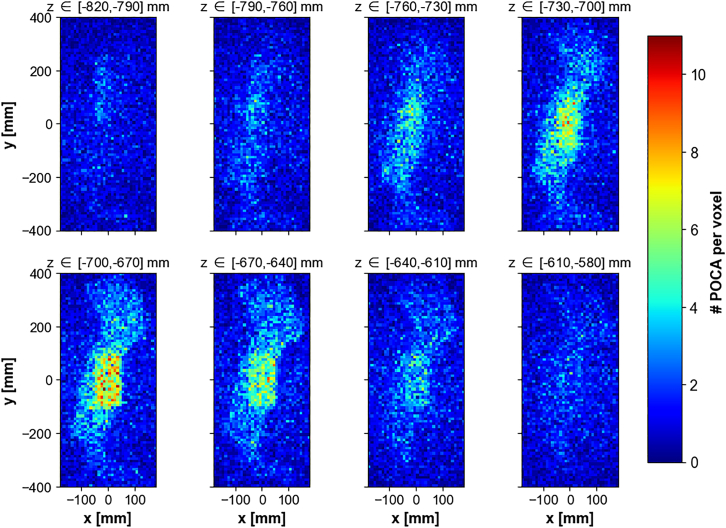
Inferred scattering density in Scenario I, computed as the number of POCA points per voxel, for a 2.6 mrad angular resolution apparatus Density predictions are shown per slice of 3 voxels layers along the vertical direction *z*, with a voxel size of 10mm.

This effect is even more visible on the slices of density predictions taken along the vertical *z* direction, as shown on Figure 7.

To address this concern, noise reduction procedures are applied at two stages of the image reconstruction process.

**Figure 8:**
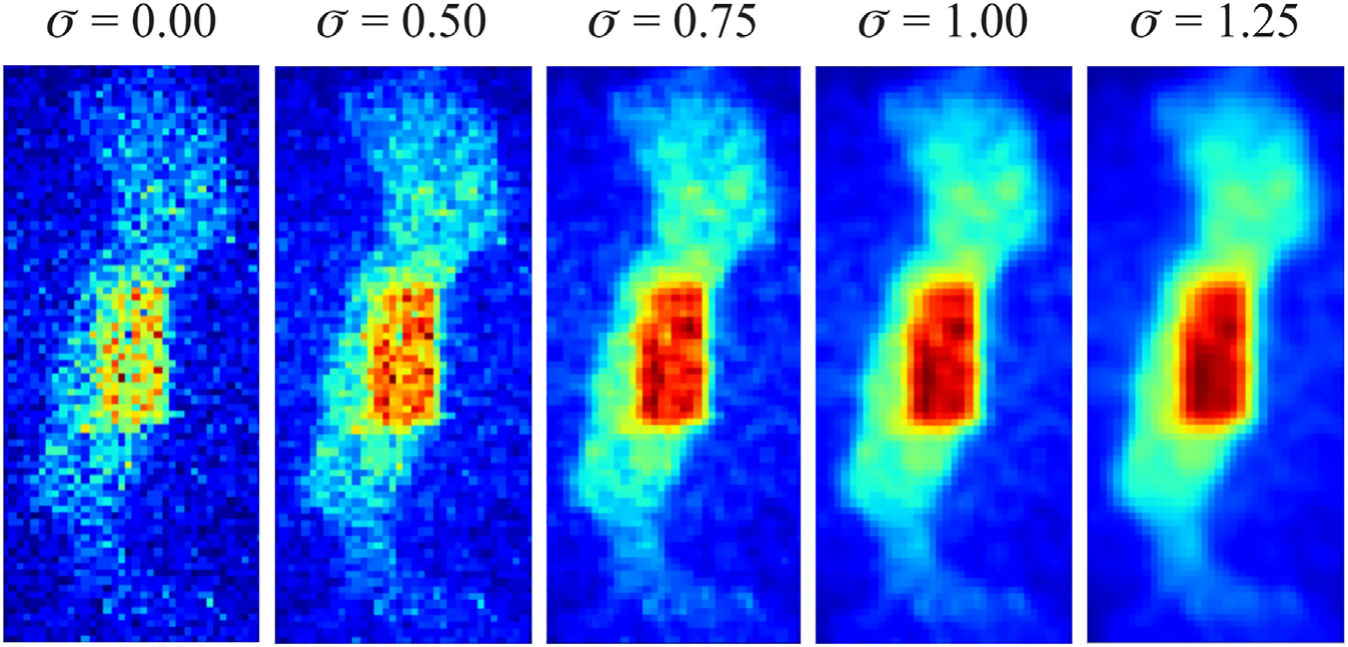
Illustration of 3D Gaussian smoothing kernels applied on voxelized scattering density predictions, for various kernel sizes *σ*

(1) Pre-processing: Because of the detection system angular resolution, straight muon tracks can be reconstructed as scattered tracks, mimicking the behavior of muons traversing dense materials. Such events are a source of noise, and are removed if their reconstructed scattering angle is below 13 mrad which corresponds to 5 times the apparatus angular resolution. While it exists a scattering angle threshold that grants an optimal signal to noise ratio, its value highly depends on the nature/density of the material and the type of imaging task.
(2) Post-processing: While many noise-reduction algorithms used in other fields like medical imaging^75^ can be adapted to 3D images, Gaussian smoother is general enough to applicable in most scenarios and have proven to be efficient in the context of anomaly detection.^76^ The 3D scattering density predictions are smoothed using a 1.5 voxel wide Gaussian smoothing kernel along the *x*, *y* and *z* direction, as described in STAR Methods. Figure 8 compares the effect of various smoothing kernel values.

**Figure 9:**
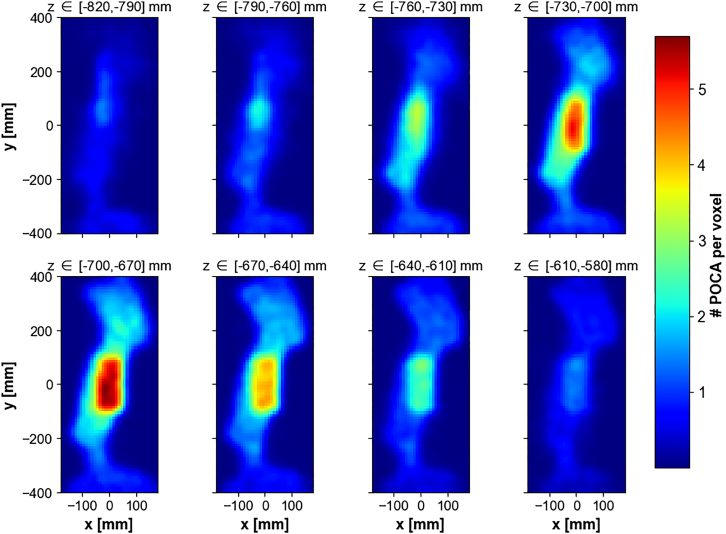
Inferred scattering density in Scenario I, computed as the number of POCA points per voxel after applying a 1.5 voxel wide Gaussian smoothing kernel and filtering out muon events with scattering angle <13 mrad Density predictions are shown per slice of 3 voxel s layers along the vertical direction *z*, with a voxel size of 10mm.

Figures 7 and 9 show the density predictions by slice of 3 horizontal voxels layers along the *z* direction, without and with these noise reduction procedures, respectively. The bronze cylinder can easily be identified, as it appears as a higher density region somewhere between z=−74cm and z=−62cm. The true location of the center of the cylinder is (x,y,z)=(0,0,−71)cm.

The reconstructed data obtained from the POCA approach consist in a collection of four-dimensional data points: the x,y,z location of the point as well as the associated scattering angle. In such a context of low-dimensional data, clustering algorithms can shine by gathering points sharing similar proprieties and thus providing a material discriminator.

**Figure 10:**
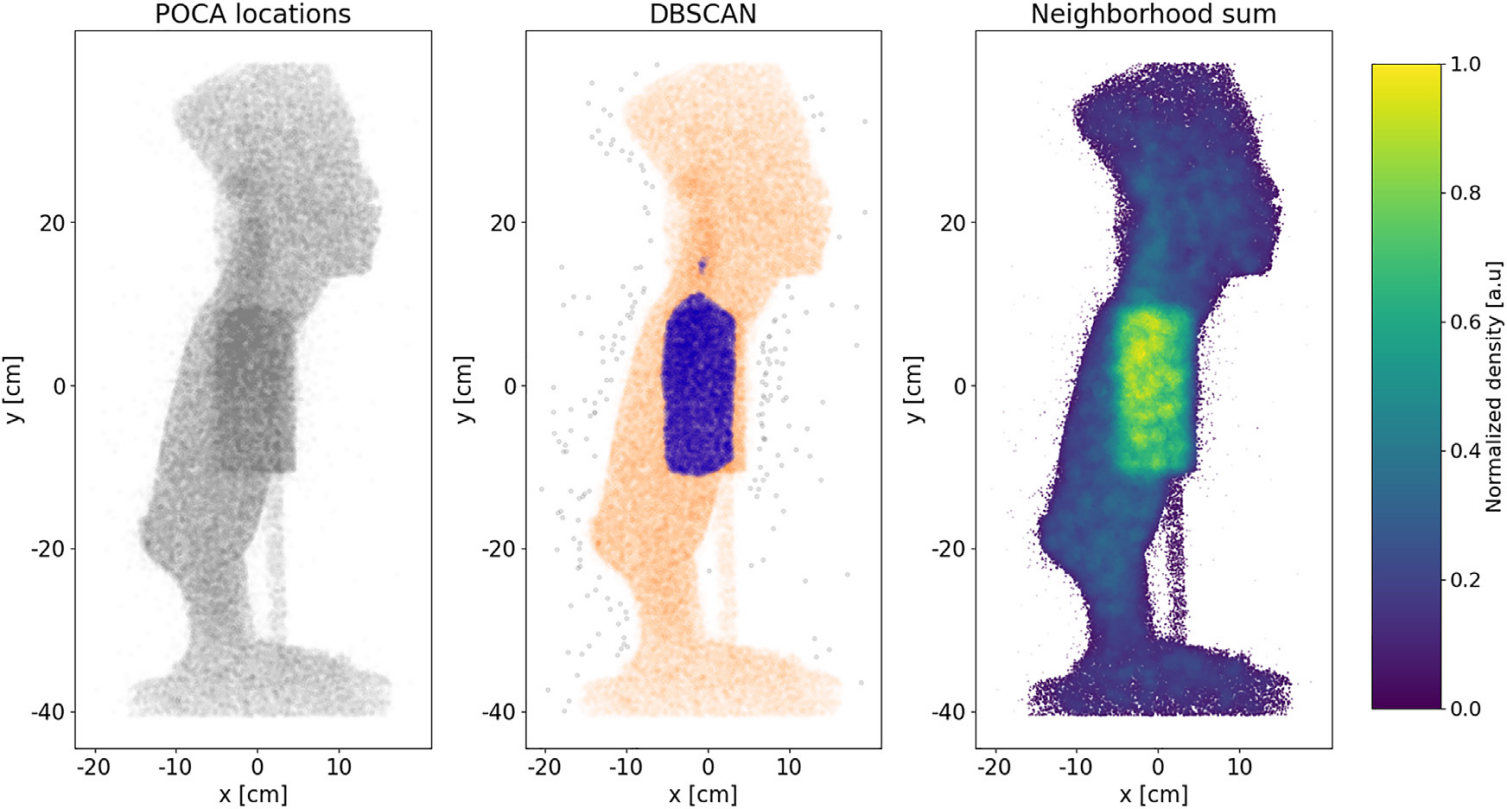
Illustration of how POCA input can be used for classification in Scenario I 2D projections are used for simplicity, although these methods work also in 3D. Left: input, i.e., POCA x,y locations (not voxelized, differently from previous figures). Center: DBSCAN classification based on POCA locations, where marble and bronze are indicated with orange and blue, respectively. Right: neighborhood sum classification based on POCA locations and scattering angles.

The DBSCAN algorithm,^77^ discussed in STAR Methods, is capable of identifying clusters of points within a dataset. These clusters are defined as areas of higher density compared to the rest of the data. In this example, the input for DBSCAN is bidimensional, consisting of the x and y position information of the POCA points (depicted in Figure 10 left), but the algorithm can be generalized to solve problems in three or higher dimensions. In Figure 10 center, a nested two-layer DBSCAN is employed. It first effectively separates objects from the noisy background POCA points (gray) and subsequently distinguishes between marble (orange) and bronze (blue) materials.

So far, in Figures 7 and 10 left and center, only the positional information has been utilized for visualization and material discrimination. However, the Neighborhood sum^78^ offers a unique clustering mechanism by incorporating both position and scattering angle information. The method’s details are presented in STAR Methods. In the current scenario, the x and y position information from the POCA points are utilized to compute the Neighborhood sum, incorporating their respective scattering angles. The Neighborhood sum information is illustrated in Figure 10 right, effectively delineating regions of higher density and high scattering angle, thereby distinguishing between marble and bronze.

### Scenario II: Average size regime

A relevant question for planning a muography investigation of an object is whether scattering or absorption is the most appropriate method. Based on which is chosen, the arrangement of detectors is rather different as illustrated in Figure 5. Scenario II is designed such to be a challenging regime for both absorption and scattering muography. Because the scattering technique requires a set of detection planes above and below the object, the overall price scales faster with the detection area than the absorption technique. The statue’s thickness being 60cm, is rather thin by absorption muography standards as muons loose only a small fraction of their kinetic energy making their absorption within the object unlikely. Both datasets use similar detection set up made of $1\;m^{2}$ detection panels.

#### Scattering muography

**Figure 11:**
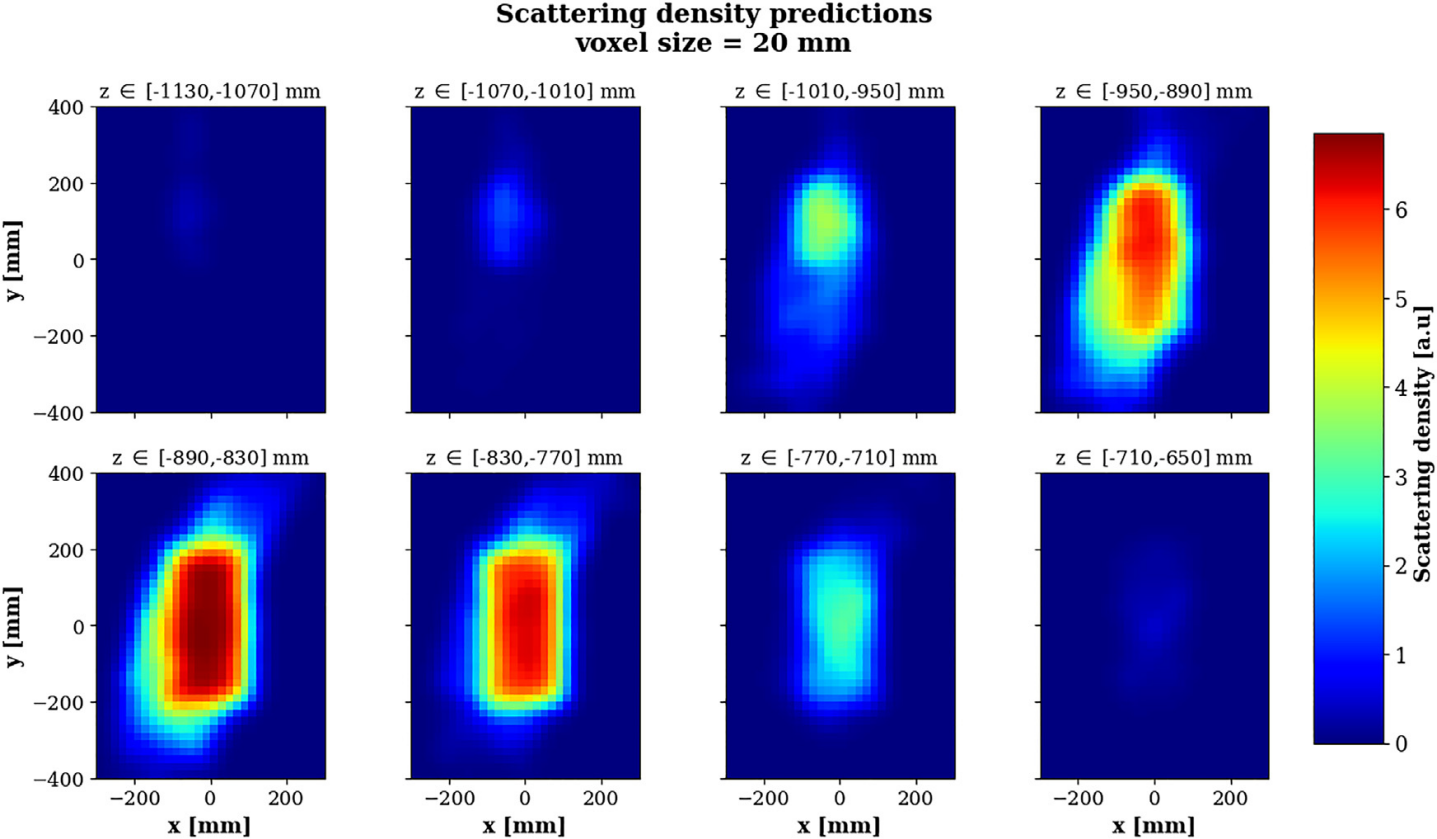
Normalized density predictions for the scattering technique using the Binned Clustered Algorithm, for Scenario II Predictions are shown by slice of 3 voxels layers along z (vertical axis).

Similarly to scenario I: small size regime, density predictions are obtained from the POCA locations. Additionally, the Binned Cluster Algorithm (BCA) was used,^79^ which incorporates a clustering algorithm utilizing the recorded values of the muons’ scattering angle. BCA tends to outperform simple POCA when the measured scattering angles associated to the POCA locations are large enough, therefore it is more appealing for this scenario than for the low size regime as the average scattering angles are larger for longer pathlengths. To mitigate statistical artifacts, the density predictions are then smoothed using a Gaussian smoother and are presented on Figure 11.

#### Absorption muography

**Figure 12:**
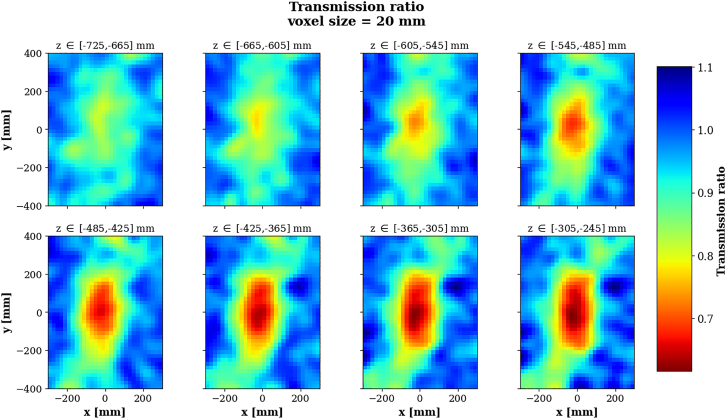
Normalized density predictions for the absorption technique and using the back projection algorithm, for Scenario II Predictions are shown by slice of 3 voxels layers along z (vertical axis).

The voxel-wise density predictions *ρ* are computed as the ratio between the recorded muon flux through the object and the free sky flux $\rho =\frac{N_{\text{object}}}{N_{\text{freesky}}}$. Both $N_{\text{object}}$ and $N_{\text{freesky}}$ are estimated using a custom back-projection algorithm inspired from^80^ and,^81^ which is further described in STAR Methods. The thickness of the object being rather small compared to absorption muography standards, it is rather unlikely for a muon to stop within the object. In order to compensate for this effect only low energy muons with kinetic energy $E_{\text{kin}}<1\text{GeV}$ were selected. Even though the detection setup used does not allow to measure the muons’ kinetic energy, a few techniques are available in the literature, see Momentum measurement. In this study we assume that the absorption-based momentum filtering method is used,^82^ being cheap and effective. Density predictions are also smoothed using the aforementioned Gaussian smoother procedure and are presented on Figure 12.

#### Scattering vs. absorption

**Figure 13:**
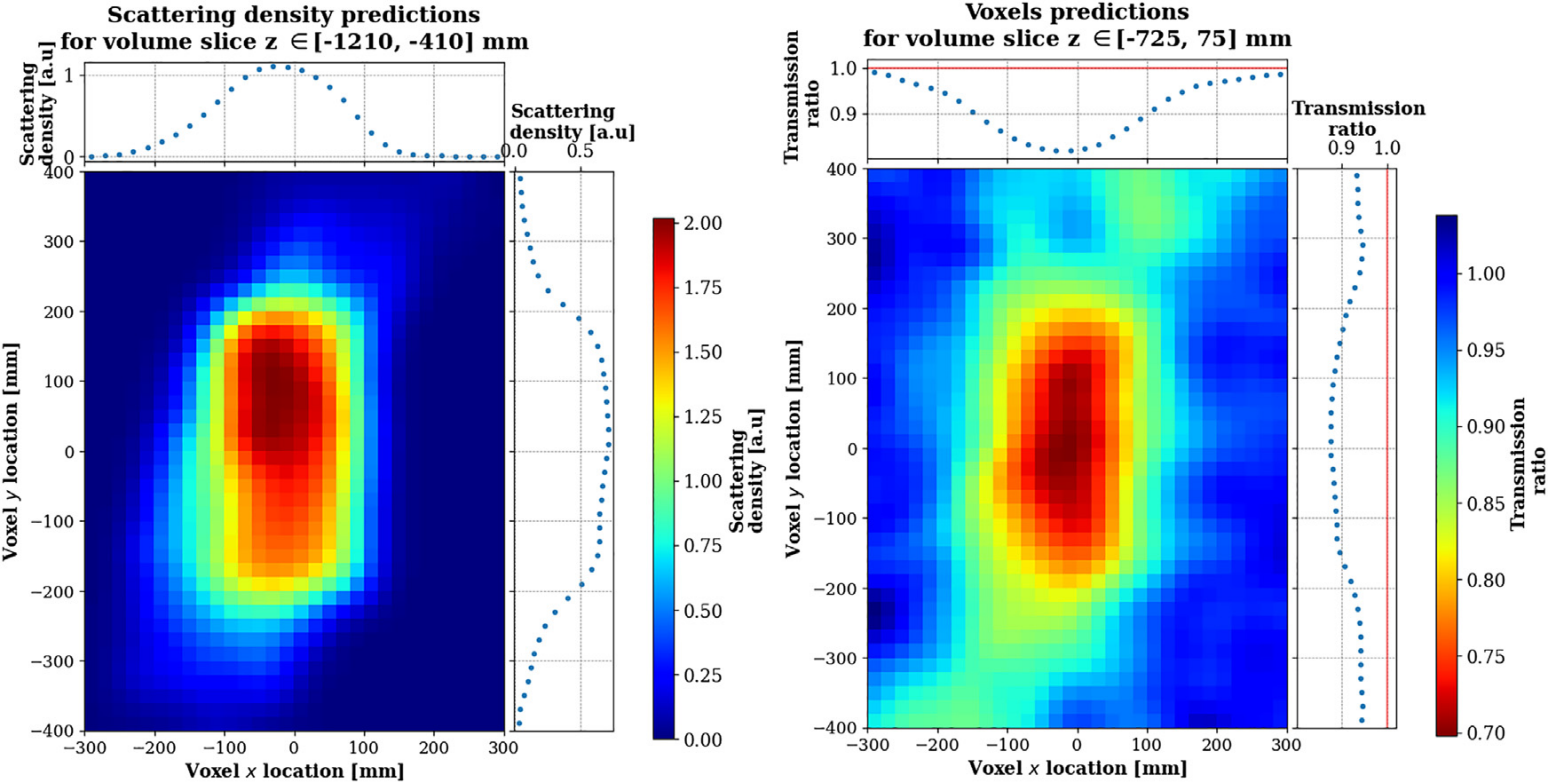
Normalized density predictions for Scenario II Left: scattering. Right: absorption. Predictions are shown by slice of 3 voxels layers along z (vertical axis). The true locations of the 20 cm diameter cylinder are respectively x,y,z,=(0,0,−810)mm and x,y,z,=(0,0,−310)mm.

Figure 13 compares scattering and absorption reconstruction for the volume’s slice corresponding to the actual position of the bronze cylinder. The presence of a high-density material can be clearly identified. Scattering clearly outperforms absorption given that it is sensitive to marble. Absorption predictions are noisier but such effect can be mitigated by increasing the acquisition time.

### Scenario III: Large size regime

This section aims at demonstrating that the use muon tomography based on muon absorption can be meaningful in the context of water content assessment, as elaborated in moisture content measurements.

**Figure 14:**
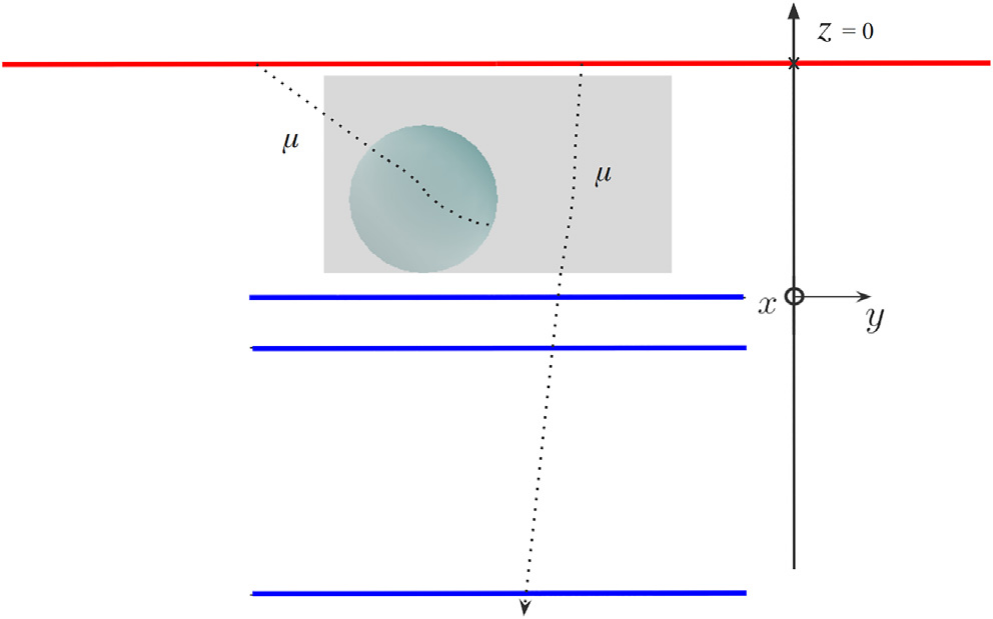
Geant4 simulation setup for the third scenario The horizontal surface from which the muons are generated is represented by the red line. The parallelepiped and spherical volumes contain calcium carbonate limestone with two different water content values.

The target volume to image is a $70\times 70\times 40\;{\text{cm}}^{3}$ block of calcium carbonate limestone with 40% porosity and 1% saturation, which translates to a 0.5% moisture content (computed from Equation 1). The exact composition that we simulated consists of $99.5\%\;CaCO_{3}$ and $0.5\%\;H_{2}O$, with the percentages given in fraction of mass. The resulting density of the limestone block is $1.63g.{\text{cm}}^{-3}$, as dictated by Equation 1. The location of the center of the block is x,y,z=0,0,−210mm. Inside the limestone block is placed a 15cm radius sphere of the same material but with a higher water content, corresponding to a 80% saturation thus a 36% moisture content. The exact composition that we simulated consists of $64\%\;CaCO_{3}$ and $36\%\;H_{2}O$, with the percentages given in fraction of mass. The resulting density of the high moisture content sphere is $1.95g.{\text{cm}}^{-3}$. The location of the center of the sphere is x,y,z=−150,0,−260mm. The simulation setup is sketched in Figure 14. As in the other two scenarios, we assume 1 mm spatial resolution and 98% panel efficiency.

In this regime, absorption becomes more relevant given the large size of the object. Muons lose more kinetic energy while propagating through denser limestone, which make then more likely to decay. As a consequence, the measured transmission ratio gets significant enough to highlight the density contrast between low and high water saturation limestone.

**Figure 15:**
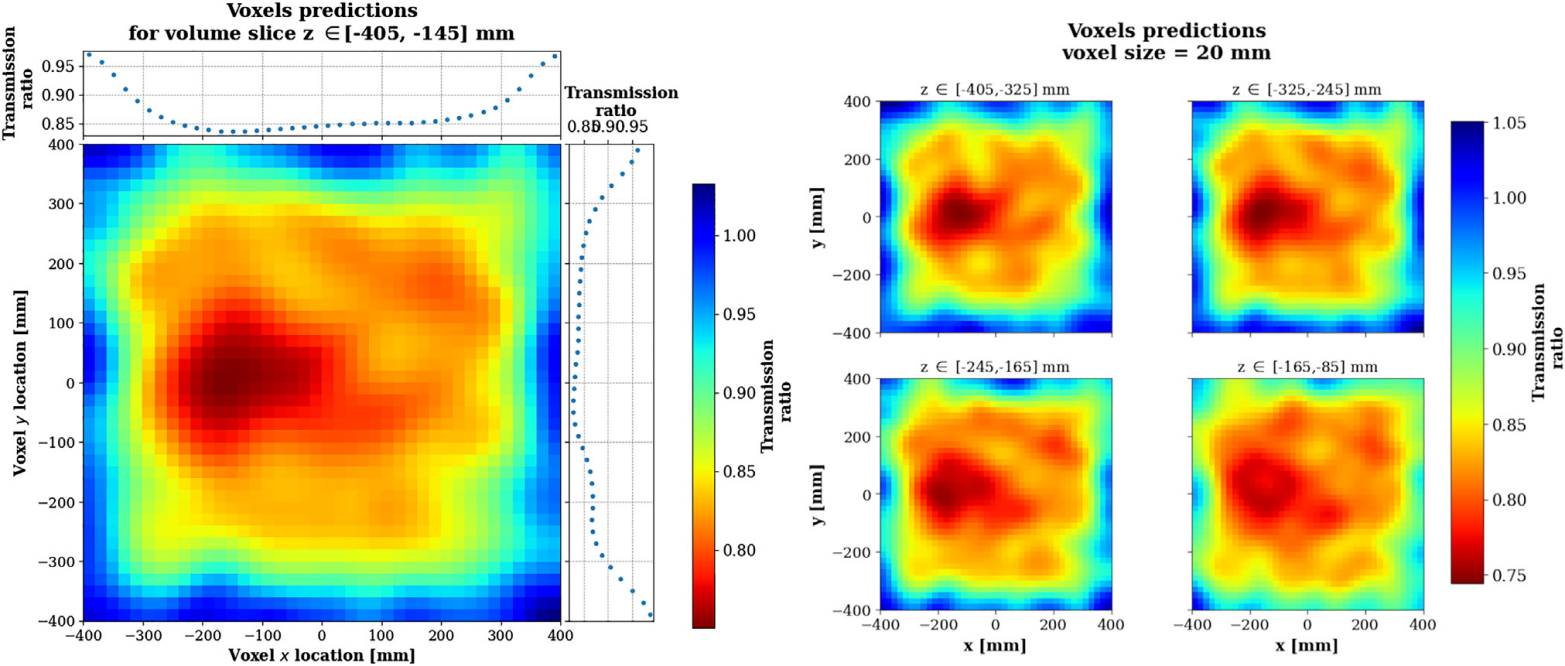
Normalized density predictions for the absorption technique using a back-projection algorithm, for Scenario III Left: projection. Right: view at four different z slices.

Density predictions presented in Figure 15 are obtained with the same back-projection and Gaussian smoothing algorithms as in scenario II: average size regime (see also STAR Methods), with a kernel size of 2 voxels.

We conclude that 8 h of data acquisition are sufficient to identify the presence of a higher density region in the limestone block. The image resolution along the x and y directions is good enough to evaluate the radius of the high water content sphere. However, the image resolution along the vertical z axis is quite poor and does not allow to precisely evaluate the z position of the sphere, as shown the left plot in Figure 15. Nevertheless, in the context of an actual measurement it is possible to combine measurements from different points of view. In this simulation, the limestone block is laid down horizontally, which is often unrealistic. In a real-life scenario, one can advantageously orient the detection system toward the target and proceed to several measurements exploiting the azimuthal symmetry of the cosmic muon flux. These various measurements can then be combined into a single 3D density map, as presented in.^83^

## Discussion

### Proposal for future case studies

This section presents three potential applications in cultural heritage where muography can make significant contributions: estimates of the moisture content, imaging of hidden features, and detection of cracks. We present possible case studies to evidence the strengths and limitations of the method, and to motivate technical or methodological improvements and needs.

#### Moisture content measurements

The ability to estimate the moisture content of sculptures or structures is important for (preventive) conservation and treatment proposals, as water is one of the main drivers for decay mechanisms such as freeze-thaw action, salt crystallization, biological growth, hygric/hydric dilation, corrosion, and reduction of strength. Sources of moisture include (wind-driven) rain, rising damp, infiltration by leakage or flooding, and condensation.

The water content, expressed as a fraction of mass (or weight percent, abbreviated in wt-%), can vary between 0 wt-% in perfectly dry conditions and 30 wt-% for various saturated porous materials (like natural stone, ceramics, mortar, wood). Typical moisture contents are in the range between 8 and 20 wt-%.

Moisture measurements can be divided in direct and indirect measurements. Direct measurements, which are the most accurate, require *in situ* sampling: the artifact is drilled to collect powder for analysis. Quantitative measurements include gravimetric weighing and drying (i.e., weighing the sample before and after drying it). For stones, a quantitative assessment can be done by the calcium carbide test, in which the moisture content of the powder from drilling is determined based on the chemical reaction $H_{2}O+CaC_{2}\to CaO+C_{2}H_{2}$ where the production of acetylene ($C_{2}H_{2}$) gas increases the pressure in a closed container. The main drawback of sampling-based techniques is their destructive nature; moreover, the calcium carbide test may underestimate the moisture content because of incomplete chemical reactions.

Indirect measurements of moisture content are based on electrical resistivity, electrical capacity, microwaves, radar, thermal imaging, equilibrium methods and time-domain reflectometry.^84^ Their main strength is that they are non-destructive. However, they face several complexities; most probe only shallow depths (of order mm to cm), some are affected by the salt content and all are dependent on the contact between probe and material. Additionally, these techniques are generally considered as merely indicative of the actual moisture content, and quantification needs time-consuming calibrations per material.^85^^,^^86^

In this context muography can find a strong use case, being an *in situ* but non-destructive remote-sensing technique whose data are directly sensitive to density and, through that, to moisture content. Indeed, the average density of the affected volume increases when wet, i.e., pores that would normally contain air would fill with water. In cases where the volume is not altered and only mass is changing (as in the case of stones), adding moisture to a stone object of density $\rho_{dry}$ results in a new apparent density $\rho^{\prime }$ according to:

(Equation 1) $$\rho^{\prime }=\rho_{dry}\times \left(1+\text{MC}\right),\text{MC}=\frac{\rho_{water}}{\rho_{dry}}\times porosity\times saturation$$

**Figure 16:**
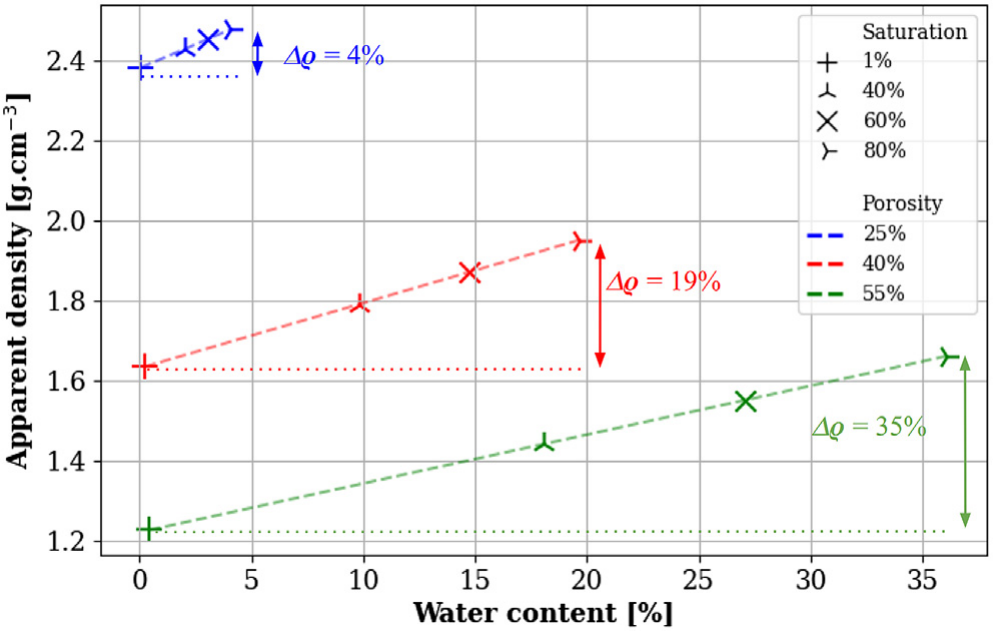
Apparent density of limestone with various porosity values (in % of the material’s volume) as a function of the water content (in % of the material mass) for different levels of water saturation (in % of the material’s mass) The relative apparent density change between 1 % and 80 % saturation (Δρ) is defined as $\left(\rho_{80\%}-\rho_{1\%}\right)/\rho_{1\%}$.

The moisture content (MC in wt-%) is expressed as the mass-fraction of water with respect to the dry material mass, i.e., $M_{water}=\text{MC}\times M_{dry}$. The porosity is defined as the ratio of the pores volume over the stone volume [vol.%] and the saturation as the ratio of the volume of water over the pores volume [%]. Figure 16 shows how the apparent density of limestone with various porosity values is affected by water saturation. For highly porous limestone with dry density $\rho_{dry}=1.22$ ${g.\text{cm}\;}^{-3}$,^87^ the change in density from a 1 % to a 80 % saturation translates to a 35 % relative change in apparent density.

In another relevant example for cultural heritage preservation, we may have a density of $\rho_{healthy}=0.8g/cm^{3}$ for healthy construction wood at normal moisture levels (around 12%), increasing to $\rho_{unhealthy}=0.94g/cm^{3}$ if a moisture pathology brings the moisture content up to 30 wt-% (This value corresponds to the average fiber saturation point of wood, at which in theory the decay will start. In practice, staining or mold growth can happen also at lower MC. In fact, decay kicks off when a liquid water source is available to the fungus, and some fungi are capable of transporting water from a distance.). Such a *ρ* difference is potentially within reach for muography.

**Figure 17:**
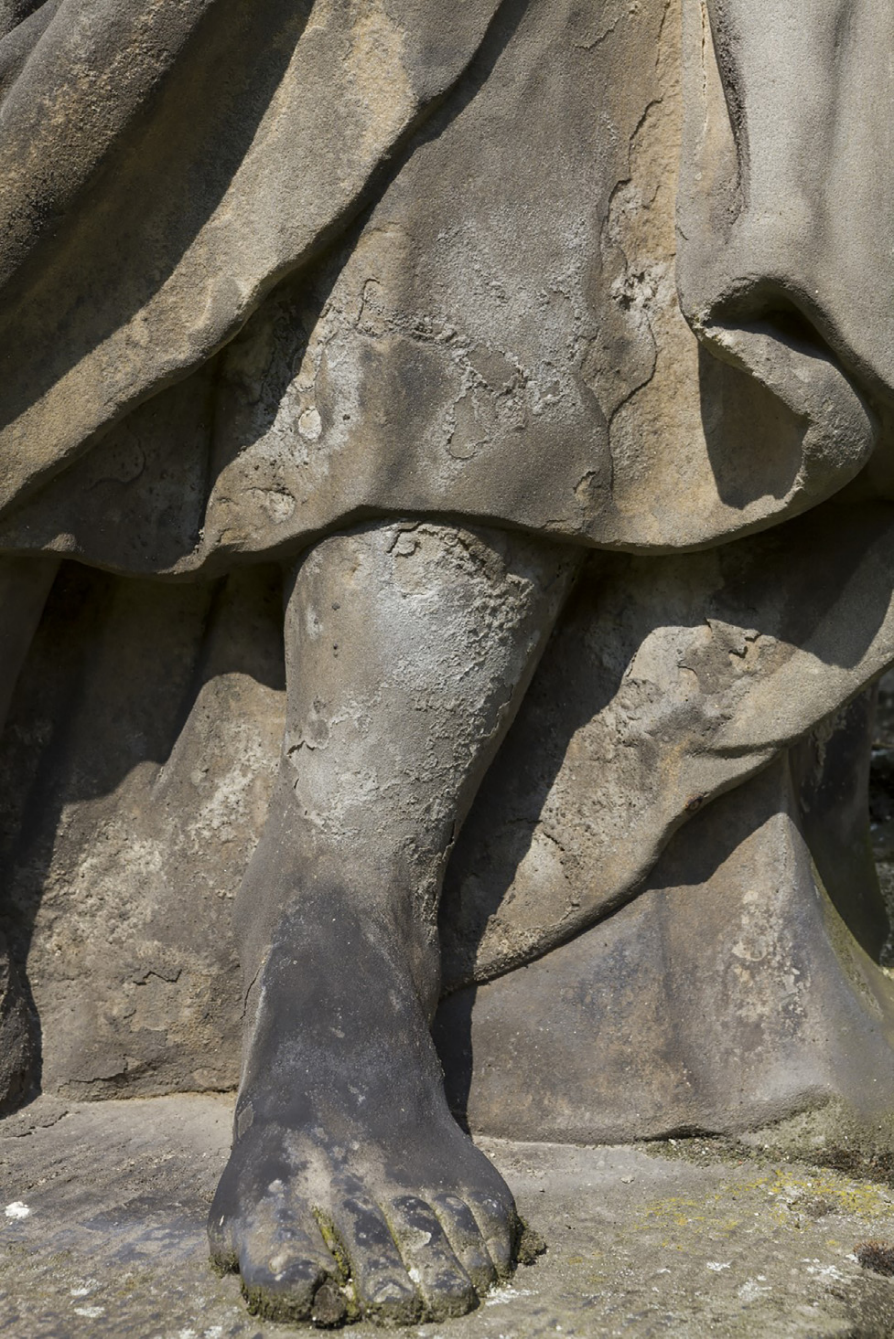
A detail of a statue with moisture and salt damage from the Calvary of St Paul’s church in Antwerp. Picture is ©KIK-IRPA, Brussels

The moisture content is dynamic, following wetting and drying, and it is a suitable problem for long-term (seasonal) muography monitoring, meaning that the observed muon flux through the monument would be plotted as a function of time. From the perspective of cultural heritage conservation, the most severe issues occur with permanent high moisture content, implying that even infrequent muography measurements would yield valuable information. A relevant case study is offered by the 68 sandstone sculptures of the Calvary of the St. Paul’s Church in Antwerp (Belgium), several of which suffer various forms of degradation attributed to the action of moisture,^88^ as shown in Figure 17.

It can be noted that spatial resolution is not an important consideration for this particular use case, differently from most other use cases discussed in this paper, as moisture spreads broadly. Moreover, to some extent it is not important to have 3D imaging, as 2D projections can already be very informative. On the other hand, precision is requested on the flux measurement, as it gets translated into an opacity measurement (where opacity indicates the integral of *ρ* along a line of sight),^20^ which is in turn used to estimate the moisture load and, if sufficient data are taken, its dependence on time.

#### Bulk features in monuments and artwork

Bulk or inner features in monuments and artwork are typically not directly observable from the surface of objects. However, these contain crucial information on construction technology, interventions, and material condition. Therefore, their identification is an essential, but not always evident part of the condition assessment of objects. Direct assessment of the bulk features requires dismantling of the structure, which is often not possible or at least risky, or destructive drill coring. Therefore, indirect and portable methods are valuable alternatives.

Muography offers the possibility to visualize inner features of a large and dense object, thanks to the high penetrating power of muons. This is particularly appealing for cultural heritage objects which contain or are suspected to contain non-trivial features that are invisible from the exterior. In this vast category of use cases, differently from the moisture measurement described previously, precision is more sought on the contrast (Δρ as function of position) than on the absolute value of *ρ*. Moreover, 3D information is usually important, demanding either more than one detector taking data simultaneously or the same detector taking data sequentially from more than one viewpoint.

This is further illustrated by a few representative examples from Brussels (Belgium), chosen among those on which some of the authors have a direct and deep knowledge. All these examples are artwork made of dense stone materials, which are difficult or risky to move and because of their size not easy to image using more traditional imaging techniques. On the other hand, their size is relatively small by the standards of muography, which means that imaging can be challenging.

**Figure 18:**
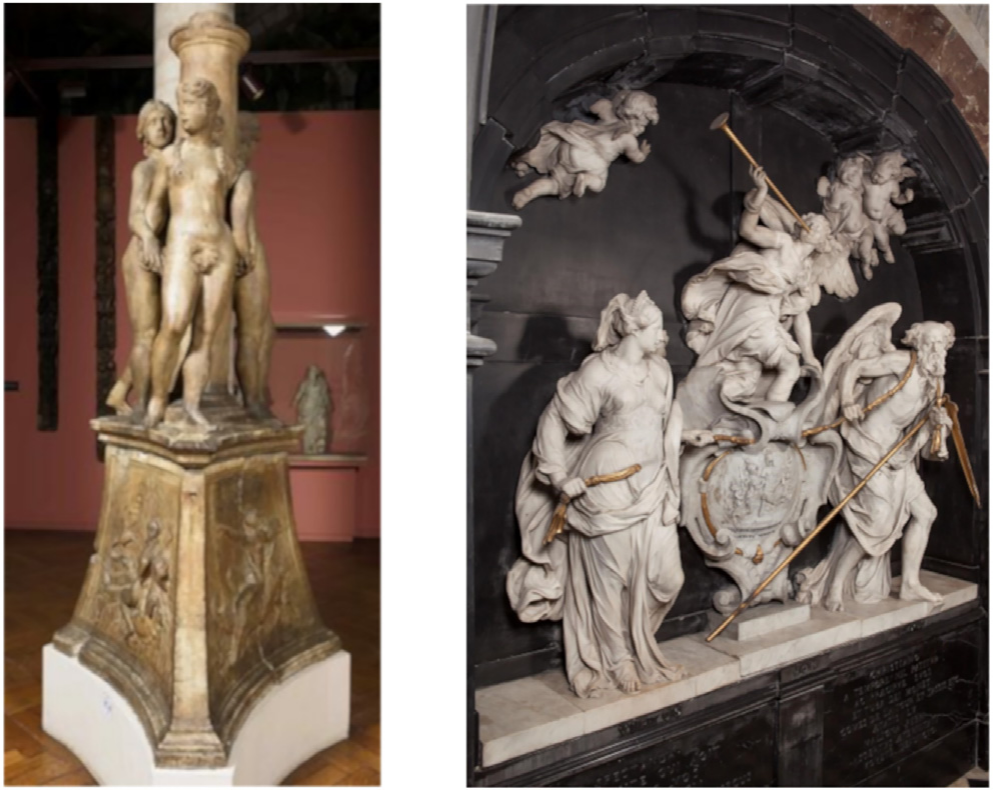
Examples of possible targets for muography Left: The Fountain of the Three Graces, in the Museum of the City of Brussels. Right: The monument of Lamoral Claude François von Thurn und Taxis, by the sculptor Mattheus Van Beveren. Pictures are ©KIK-IRPA, Brussels

The Fountain of the Three Graces (Figure 18, left), in the permanent collection of the Museum of the City of Brussels, was sculpted in white Carrara marble in the 1530’s.^89^ The fountain is composed of two main volumes of white marble: the three Graces with the central column, and the triangular base with mythological scenes. We have no information about the inner tube system that is probably still present in the core center of the column and most likely connected to the nozzles in the breasts of the female figures and the nozzle in one of the mythological scenes on the base.

The monument of Lamoral Claudius Franz von Thurn und Taxis (Figure 18, right), located in the Church of Our Blessed Lady of the Sablon (Brussels), was sculpted by Mattheus Van Beveren in 1678 in white marble with black marble cladding.^90^ The structure with black marble cladding underneath the sculptures is deformed due to subsidence, and demands further investigation of the inner brick structure to evaluate its condition. As dismantling of the sculptures poses risks for their preservation, non-destructive imaging of the structure would help in the assessment.

**Figure 19:**
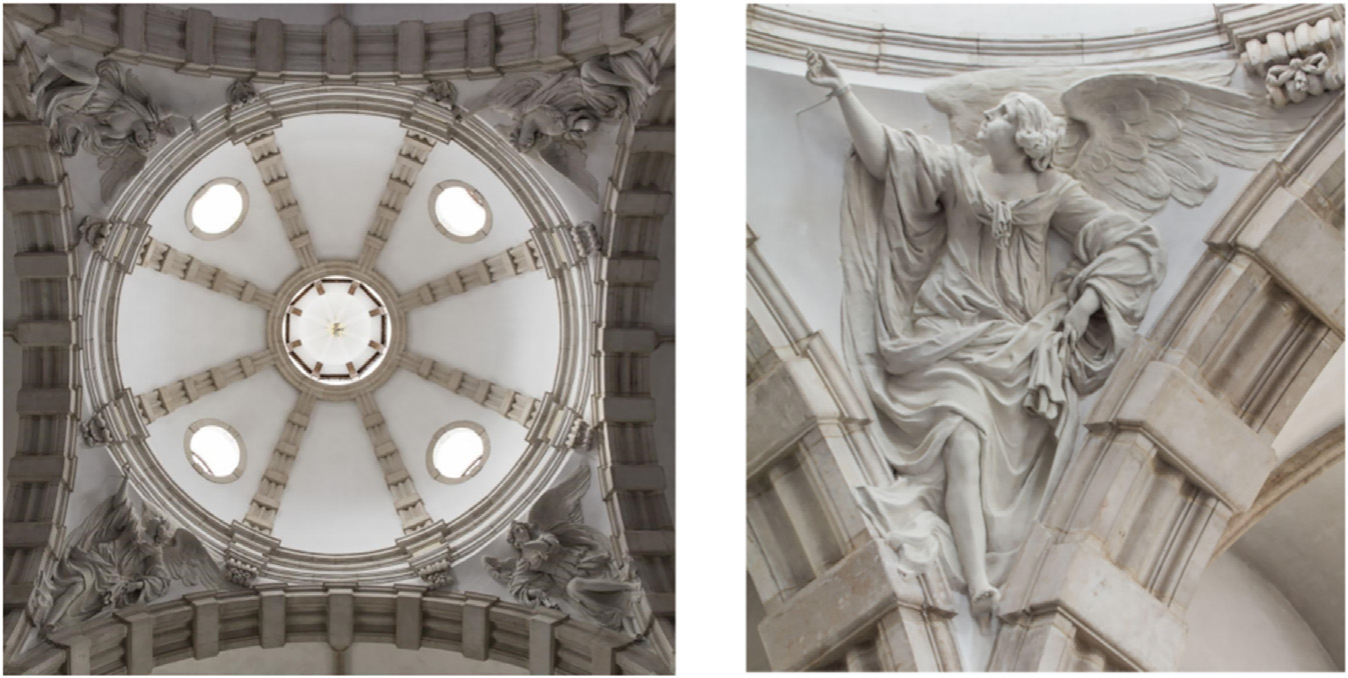
Examples of possible targets for muography Left: Four sculpted angels in the pendants of the Église Notre-Dame aux Riches Claires. Right: close-up of one of the angels. Pictures are ©KIK-IRPA, Brussels

The pendants of the Notre-Dame aux Riches Claires church in Brussels (Figure 19) host four sculpted angels, composed of different volumes of Avesnes limestone, fixed in masonry of the pendants underneath the central dome of the church.^91^ After a devastating fire in 1989, the sculptures were thoroughly restored. Unfortunately, these interventions were not documented in detail. In 2020, after the left arm of one of the angels fell down, a survey discovered several new cracks and fractures on all the four angels. Archival investigation of the work reports from the 1990’s found mention of injections with a cement based mortar and pinning of fragments by iron threaded bars/dowels to the underlying structure of the vaults, but not their exact number and positions, thus impeding to assess if the current cracks and bad condition have anything to do with those interventions, which is an extremely important question from the point of view of cultural heritage restoration.^92^ Besides that, the angels were composed of different limestone blocks. The joints are slightly visible on the surface but it was not very clear how many blocks were used (some parts are made out of lime mortar as well and during restoration, new volumes were added with a mixture of plaster and stone dust). Moreover, the original anchors and the new added dowels would be interesting to localize.

**Figure 20:**
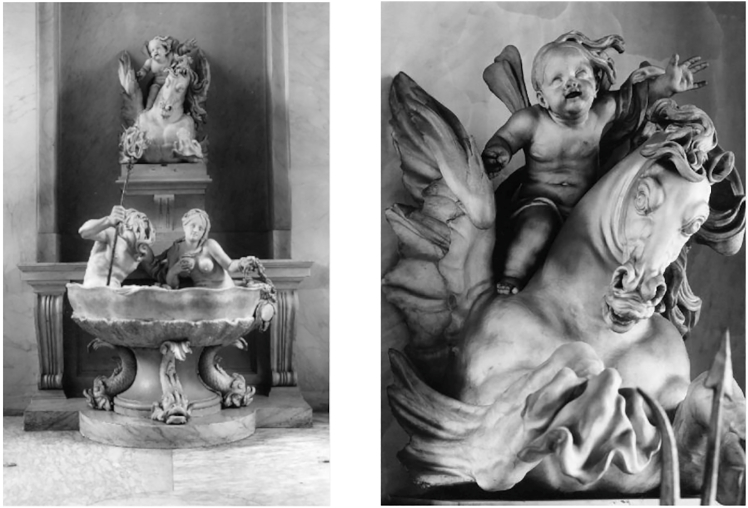
Examples of possible targets for muography Left: Fountain of the sea gods, at the Museum of Fine Arts in Brussels. Right: close-up of one of the elements of the fountain. Pictures are ©KIK-IRPA, Brussels

The Fountain of the sea gods (Figure 20) was sculpted in white marble by Gabriël Grupello in 1676^93^ and it is currently displayed in front of a staircase in the Museum of Fine Arts of Brussels. The fountain has an internal structure that would undoubtedly be interesting to investigate in view of its conservation treatment. As a matter of fact, traces of corrosion are visible on the surface of some parts of the fountain. The fountain cannot be moved, thus making a case for portable muography detectors.

#### Inner cracks

Imaging a statue or another cultural heritage item may also be motivated by searching for cracks or other forms of defects that may appear due to material degradation. This can be challenging for muography, as in practice the crack would appear as a narrow zone of lower density. Therefore, for this goal, 3D spatial resolution must be pushed to the limit.

**Figure 21:**
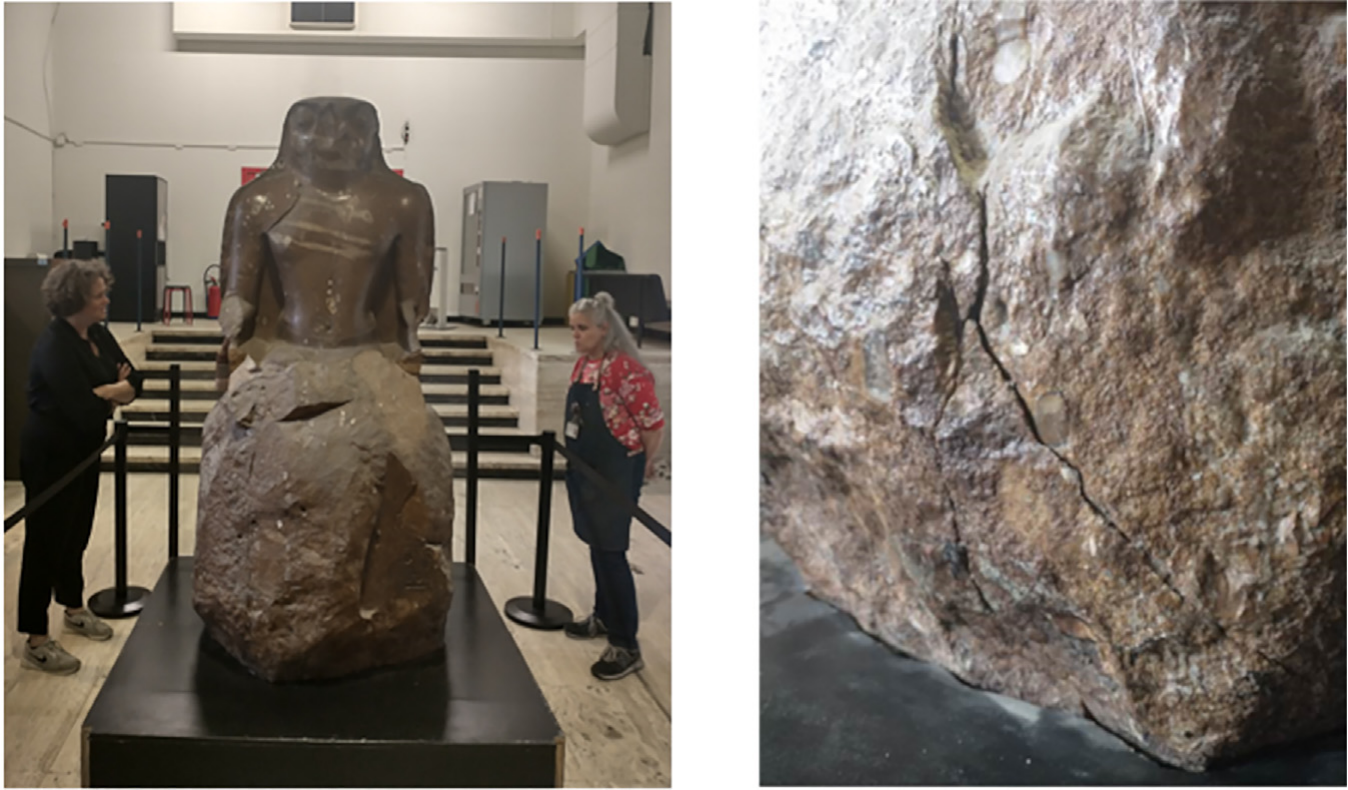
Examples of possible targets for muography Left: Sculpture of the Egyptian deity Khonsou, at the Art & History Museum of Brussels Right: a close-up of one of the visible cracks. Pictures are ©KIK-IRPA, Brussels.

As an example, Figure 21 shows a quartzite sculpture of the Egyptian deity Khonsou, dating from the 21st dynasty (1050 BCE), at the Art & History Museum in Brussels.^94^ This sculpture was seriously damaged due to a fire in the museum in 1946. Thereafter, the sculpture was stored under an open shelter where it was exposed to frost and bad weather for a long time, causing many cracks and fractures. Due to its weight (estimated around 2500 kg) and its fragility, it is not recommended to move this object in this state of preservation. Yet the museum wants to move the sculpture for renovation works and a redesign of the collection. A well-defined image of the inner fractures is necessary to evaluate its condition before conservation treatment and/or transport.

Muography is recently starting to be considered as a promising non-destructive technique for searching for cracks, in the context of civil engineering, motivated by the monitoring of aging infrastructure (e.g., bridges). A proof-of-principle experiment has been reported in ref.,^95^ based on muon scattering. For the same purpose, transmission-based muography has also been investigated.^21^ For a non-transportable item of the size of the statue described above, the latter appears to be more indicated, although the former is found to achieve a better spatial resolution.

#### Summary of proposed case studies

Table 1 summarizes the muography requirements for the typologies of case studies proposed in this section. Although at this stage these considerations are purely qualitative, they can help guide the strategy for detector development and deployment.

**Table 1:** Muography requirements for three categories of cultural heritage applications

Objective	Absolute *ρ*	*ρ* vs. position	*ρ* vs. time	Material id.
Moisture	Important	Low resolution	High frequency	Not needed
Bulk features	May be useful	Medium resolution	Not needed	May be useful
Inner cracks	Not needed	High resolution	Low frequency	Not needed

When absolute density measurements are important, as in the case of estimating the water content, it is necessary to also collect an auxiliary sample of muons under the same conditions but without the object of interest, in order to extract the transmission fraction from the ratio of the two datasets. However, in most cultural heritage use cases this is not crucial, as one is more interested in spatial resolution (hence *ρ* as a function of position) or stability (hence *ρ* as a function of time). In those cases, *ρ* only needs to be expressed as a difference or a ratio with respect to a reference. In all cases, contrast between different *ρ* values is important.

Spatial resolution in the measurement of *ρ* vs. position is always somewhat important when the feature being sought is a cavity or a crack, or a region of different material. Small features, including cracks, are challenging for absorption muography, therefore scattering muography with its superior intrinsic resolution should be preferred whenever it is realistic for the object to be sandwiched between muon detectors. Scattering muography is also to be preferred to absorption when the different material of the inner feature happens to be similar in density to the bulk material.

Moisture content and inner cracks are two examples where it may be important to monitor the evolution of the object with time, in order to intervene at the first indications of a dangerous degradation of the conditions. The water fraction of an object can change quickly with time (especially outdoors), justifying in some cases a continuous monitoring, while the monitoring of cracks may be performed with infrequent data-taking campaigns.

### Conclusions

Muography is a promising tool due to its relatively low cost and portability, given the inherent availability of the cosmic muons that constitute its “radiation source”. Thanks to the large muon penetration power, it is complementary to other imaging methods. We argue for the potential interest of muography as a nondestructive subsurface imaging technique in several use cases in cultural heritage conservation.

In this paper, we reviewed the state of the art in portable techniques for cultural heritage analysis and in muography applications to large-size cultural heritage and we argued for applying the same technique to some use cases characterized by a smaller size (human size or smaller), which is a regime so far under-explored in muography. To assess the regimes of interest for muography, we reported a simulation study in ideal conditions with both scattering and absorption muography, exploring the strengths and limitations of both techniques for statues with different size.

Absorption and scattering have complementary strengths and weaknesses, but some limitations are in common for both: long acquisition times are necessary, due to the relatively low natural rate of cosmogenic muons, and muon direction and energy cannot be controlled. In spite of those limitations, both techniques can provide unique data in various use cases, which we have qualitatively described with specific examples from our local context, which may become concrete targets for future muography studies. Targeting density differences between different inner parts of the object of interest, or searching for cracks, are typical use cases where the portability of muography detectors is appealing when the object of study is too fragile for transportation. Human-sized statues, however, appear to be at the lower limit of sensitivity of muography, motivating significant development work on precise instrumentation and cutting-edge algorithms for image reconstruction. Moisture load measurement might be another promising use case for muography. To the best of our knowledge, this has not been exploited yet in the relevant literature.

### Limitations of the study

A limitation that is unavoidable in muography, in any of its variants, is that long acquisition times are necessary due to the relatively low natural rate of cosmic-ray muon production. Moreover, in contrast with most other particle-based methods, direction and energy of these muons cannot be controlled. (Some possible implications of controlled and intense muon beams for absorption- and scattering-based muography are discussed in.^25^ Note, however, that some drawbacks would be in common with the MIXE technique discussed in the introduction). For these reasons, it is difficult to expect muography to out-compete the precision of more traditional techniques, in the regimes of applicability of the latter: In this paper, we argue for applications of muography in use cases where any other technique is unviable or discouraged. In such use cases, no experimental data are available yet.

Simulation studies such as those presented in this paper are only meant as orientation for further research, but real-world applications might face practical constraints that are hard to simulate. For example, the absorption method relies crucially on the ratio of the flux measurement through the object of interest over a free-sky reference. The implicit assumption is that the only significant difference between the two datasets is the presence or absence of the object, and any other confounding factor affecting the flux would cancel away in the ratio. But there are different ways to collect a free-sky reference sample, each with its own drawbacks. The same muon detector may be used to take data twice, pointing first toward and then away from the object of interest; in principle, this ensures that dishomogeneities in the detector response cancel out, but on the other hand, both the muon flux and some detector parameters may be sensitive to time-dependent cosmic or environmental effects.^20^ Alternatively, the regular and free-sky datasets can be recorded simultaneously with different detectors pointing in different directions, as in.^96^ One method or the other is to be preferred depending on the trade-off, that is hard to predict, between the stability in time of the flux measurements and the reproducibility of the detector behaviors.

A practical consideration that is not often discussed, but on which the success of muography stands or falls, is the cost of the equipment. A rough comparison of costs for typical muography detector technologies has been presented in.^20^ At the present state of the art, it is unfair to compare the costs of muography setups with those of the equipment needed in the standard methods reviewed in state of the art in portable non-destructive test methods for cultural heritage, as the latter are so widespread to be mass-produced and usually available off-the-shelf. In contrast, muography is still in a pioneering stage, where most detectors are individually built and optimized by small teams. Some of the authors of this article have shown in ^97^ how to build a small muon tracker with reasonable performance using spare components and cheap additional materials and consumables; but this relied on the availability of a well-equipped particle physics laboratory, while the set-up of a suitable detector-building facility from scratch entails very large initial costs. On the other hand, costs would be brought down by large factors if muography becomes commercially viable and reaches the point of mass production. Considerations related to commercial prospects of muography have been discussed in^98^; the same article also argues that mobile detector systems will become more and more common in the future, which is particularly relevant for the applications discussed here.

## Resource availability

### Lead contact

Requests for further information and resources should be directed to and will be fulfilled by the lead contact, Andrea Giammanco (andrea.giammanco@cern.ch).

### Materials availability

This study did not generate new materials.

### Data and code availability

#### Data

Only simulated data are used in this study. All simulated datasets used in this study can be reproduced by running our code (see next paragraph) following the instructions in its “README” file. In some of our examples (scenario I: small size regime and scenario II: average size regime) we made use of micro-CT data privately provided by UGent-Woodlab and the Royal Museum for Central Africa (Cultural Anthropology and History Department), produced in the scope of the ToCoWo project. Access to those data can be requested to the ToCoWo team, as indicated at their website https://tocowo.ugent.be/.

#### Code

All original code for simulations and data analysis, as well as the configuration data for the external software tools that are necessary to reproduce the datasets (CRY,^66^ GEANT4,^67^ B2G4^72^) and the results presented in results, are publicly accessible at the URL https://github.com/MaximeLagrange/muograph.

#### Other items

Any additional information required to reanalyze the data reported in this paper is available from the lead contact upon request.

## Acknowledgments

We thank KIK-IRPA for the figures in proposal for future case studies. UGent-Woodlab and the Royal Museum for Central Africa (Cultural Anthropology and History Department) are acknowledged for providing Figure 4 and micro-CT data of that statue in the scope of the ToCoWo project (https://tocowo.ugent.be/). The ToCoWo project is funded by the 10.13039/501100002749Belgian Science Policy Office (BELSPO; B2/191/p2/TOCOWO). Jan Van den Bulcke of UGent-Woodlab provided helpful feedback on our considerations about estimating moisture content in wood.

A preliminary version of the simulation study reported in results has been presented at the Muon4Future workshop and in the corresponding proceedings.^25^ We are grateful to several participants at that workshop for their constructive feedback.

This work was partially supported by the Fonds de la Recherche Scientifique - FNRS under Grants No. T.0099.19 and J.0070.21, and by the EU Horizon 2020 Research and Innovation Programme under the Marie Sklodowska-Curie Grant Agreement No. 822185.

## Author contributions

All authors contributed to writing and reviewing the manuscript. In addition, M.A.M., M.L., and V.K. produced and analyzed the datasets and developed original reconstruction algorithms; M.B., T.D.K., J.D.R., and S.H. critically reviewed the state of the art in portable non-destructive test methods for cultural heritage, and proposed suitable case studies for muography; A.G. coordinated the project as a whole and the editing of the manuscript, supervised the simulation and analysis of data, and critically reviewed the state of the art in muography.

## Declaration of interests

The authors declare no competing interests.

## STAR★Methods

### Key resources table

**Table:** 

REAGENT or RESOURCE	SOURCE	IDENTIFIER
**Deposited data**		

Micro-CT imaging data of a wooden statue	UGent-Woodlab and the Royal Museum for Central Africa (Cultural Anthropology and History Department); data created in the context of the ToCoWo project (https://tocowo.ugent.be/)	BELSPO identifier B2/191/p2/TOCOWO

**Software and algorithms**		

Simulation and data analysis code	This paper. (Can be downloaded from https://github.com/MaximeLagrange/muograph)	Version 0.1.9
GEANT4	Agostinelli et al.^67^, https://doi.org/10.1016/S0168-9002(03)01368-8 (Can be downloaded from https://geant4.web.cern.ch/)	Version 10.07
CRY	Hagmann et al.^66^, https://doi.org/10.1109/NSSMIC.2007.4437209 (Can be downloaded from https://nuclear.llnl.gov/simulation/main.html)	Version 1.7
B2G4	Rodriguez et al.^72^, https://doi.org/10.31526/jais.2024.476	Beta version accessible, upon request to the B2G4 authors, at the URL: https://github.com/DLR-MI/B2G4

### Method details

#### Point of closest approach

The Point Of Closest Approach algorithm works by finding the closest point between the incoming and outgoing tracks. It interprets this point as where the muon likely had a single high-energy elastic interaction with a nucleus. This approach neglects actual electromagnetic interactions that occurs along the its trajectory. Even though it is a rough approximation of reality, it has proven to be effective in many applications. Specifically, when the muon’s scattering angle is large with respect to the angular resolution of the detection system, provides a good approximation for the muon scattering vertex. However, it has limitations when dealing with small scattering angles and nearly parallel incoming and outgoing tracks. Consequently, this leads to the reconstruction of POCA points outside the Volume of Interest (VOI), as illustrated in Figure 3. In situations where spatial resolution is not perfect, as much as 40 % of the POCA points can be reconstructed outside of the VOI. The problem can be somewhat resolved using an iterative POCA approach.^107^

The output of the POCA algorithm is a series of four-dimensional data points; the x,y,z locations as well as the scattering angle dθ between the incoming and outgoing tracks. Their spatial distribution reflect the density of the material and can be used as input of more advanced density reconstruction and clustering algorithms.

#### DBSCAN

The DBSCAN algorithm^77^ is one of the most commonly used clustering algorithm. It takes two parameters as input, $N_{\min}$ and *ϵ*, where $N_{\min}$ is the minimum number of points within a radius *ϵ* (an “*ϵ*-neighborhood”) to form a dense region. Starting from a random point that has not been visited, the number of points N within its *ϵ*-neighborhood is computed. If $N>N_{\min}$, a cluster is started, otherwise, the point is labeled as noise. If a point is found to be a dense part of a cluster, its *ϵ*-neighborhood becomes part of that cluster. These steps are repeated until the whole density-connected cluster is found. Then, a new unvisited point is retrieved and processed, leading to the discovery of further clusters or noise.

When applied to POCA locations, the main limitation of this algorithm lies in finding the appropriate values of *ϵ* and $N_{\min}$. Given that the distribution of POCA locations is affected by various parameters such as exposure time, material density or even the position of the volume within the detector’s acceptance, their is no such thing as an optimal parameters choice that would suit every muon scattering tomography experiment. In this study, *ϵ* and $N_{\min}$ were obtained through brute force parameter scanning.

#### Neighborhood sum

The Neighborhood Sum algorithm,^78^ adeptly combines density of scattering points and scattering angle values. This is achieved by computing the sum of scattering angles within a defined neighborhood, a circle in 2D or a sphere in 3D scenarios. Notably, this method excels in material discrimination, leveraging the dependency of scattering points and angles on material density. Regions with high density points and scattering angles are accorded higher weight, while noise points, including those with elevated scattering angles, are effectively suppressed due to the lack of neighboring points. This method performs well in situations with few scattered vertices, benefiting from efficient data aggregation to reduce reliance on individual points and enhance parameter estimation.

#### Binned clustered Algorithm

The Binned Clustered Algorithm was developed in^79^ is an extension of the POCA algorithm. It assigns to each voxel a score by taking into account the degree of spatial clustering of the POCA locations in addition to the associated scattering angle value. For each pair of POCA locations i, j in a each voxel, a metric $s_{ij}$ is computed as:

$$s_{ij}=\frac{d_{ij}}{\theta_{i}\theta_{j}}\text{,}$$

where $d_{ij}$, $\theta_{i}$, $\theta_{j}$ are respectively the euclidean distance between *i* and *j*, the scattering angles associated to *i* and *j*. Finally, the final score of each voxel is computed as the median of its $s_{ij}$ distribution. In order to reduce noise, the final score of voxels with less than five POCA locations is set to 0.

#### Gaussian smoothing

Gaussian smoothing is a commonly used technique in image processing, allowing to reduce the noise of an image. Mathematically, it corresponds to convolving the image with a Gaussian function. Each pixel value is modified to a weighted average of that pixel’s neighborhood, with weights computed as a 2D Gaussian:

$$G\left(x,y\right)=\frac{1}{2\pi \sigma^{2}}e^{-\left(x^{2}+y^{2}\right)/2\sigma^{2}}\text{,}$$

where x,y are neighboring pixel positions with respect to the central one. The above formula can easily be generalized to 3D, which makes it a interesting tool for 3D imaging techniques like muon scattering tomography as demonstrated in.^76^ Figure 8 shows the effect of 3D Gaussian smoothing kernels on scattering density predictions for various kernel sizes. The latter dictates the magnitude of the smoothing along a given direction, and should be chosen carefully to avoid over-smoothing which can be counter productive in the context of edge-detection tasks. While the illustration presented in Figure 8 utilises the same kernel size along each direction, the kernel sizes along the horizontal and vertical directions can have different values in order to account for the vertical blurring of scattering density maps caused by the verticality of the muon flux.

#### Backprojection algorithm

**Figure 22:**
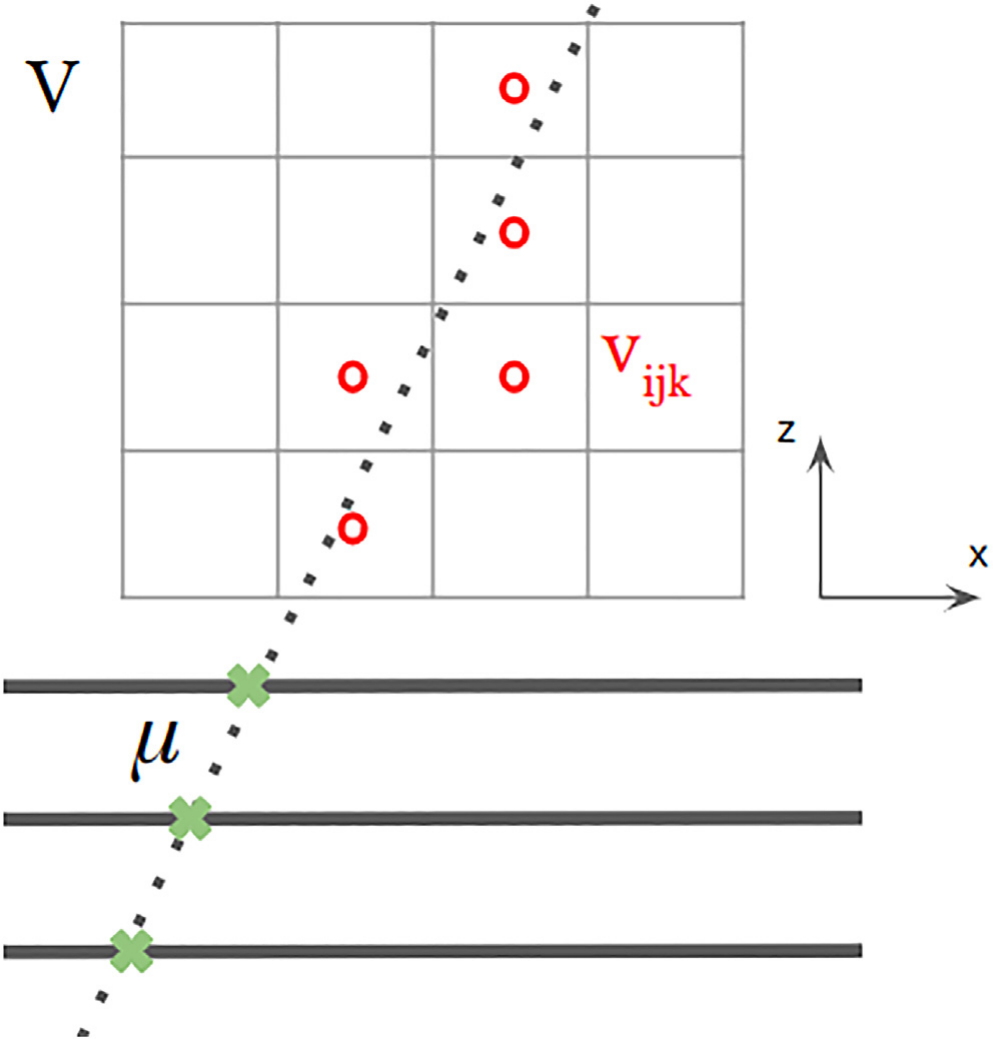
Back-projection of a reconstructed muon track into a voxelized volume of interest *V* in the context of absorption-based muography experiment The voxels $v_{ijk}$ traversed by the muon track are marked by a red circle.

As discussed in state of the art and developments of interest in muography, most of state of the art absorption-based muography experiments are dealing with large objects, i.e., from tens of meters to kilometers. The detection system being a few orders of magnitude smaller, and/or placed so far away from the target that it can be considered as a point-like object. In this context, one only measures the muon direction (θ,ϕ) and compute the transmission map as a 2D histogram with *θ* and *ϕ* bins. However, to study human-sized sculptures we have in general the possibility to position the detectors very close to the statue, in order to maximize the resolution within the object, and this approximation is no longer valid. One can use the (x,y) position of the first muon hit in the detector as an additional measurement, making it possible possible to back-propagate muon tracks into the volume of interest and obtain density information about the volume based on the number transmitted muons. In the studies of Sections 2.2 and 2.3 we perform 3D reconstruction using a fairly simple algorithm, inspired from^81^ and,^80^ and presented in Figure 22:

(1) Define a volume V within detector acceptance.
(2) Divide the volume into voxels of size *d*.
(3) For each voxel, count the number muon tracks passing through it.
(4) At the end of the scan, each voxel has a number of muons *N* associated to it.

One needs two datasets: one with free sky and one with the target object. Then the muon transmission ratio of each voxel computed as $\frac{N_{\text{object}}}{N_{\text{free}-\text{sky}}}$ can be used for estimating the object’s average density.

### Quantification and statistical analysis

There are no quantification or statistical analyses to include in this study.

### Additional resources

There are no additional resources to include in this study.

## Figures and Tables

**Figure 23:**
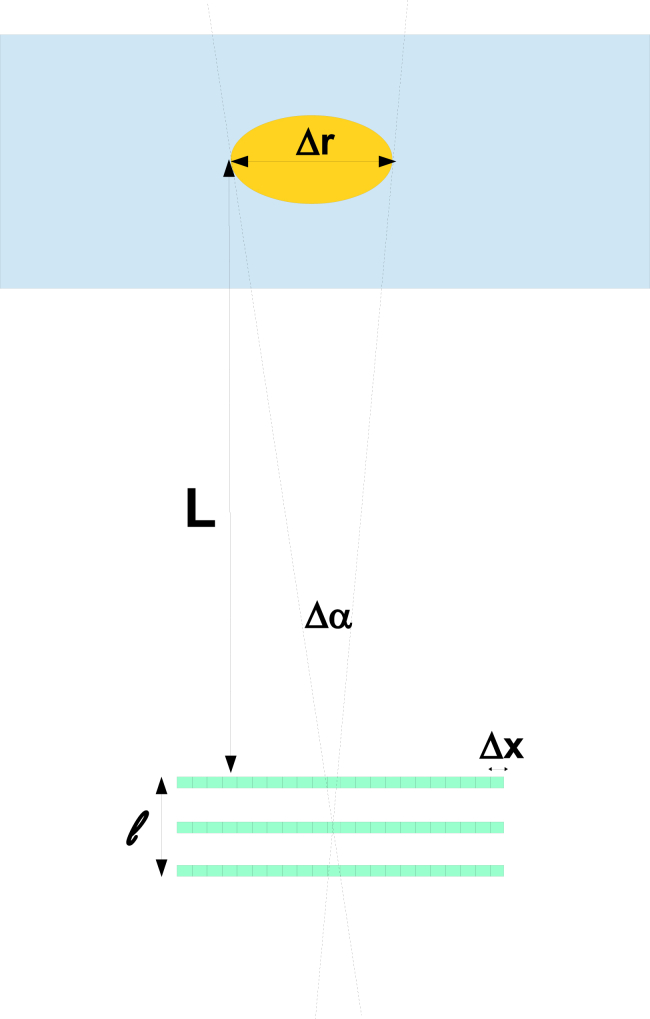
Sketch illustrating the relationship between the resolving power Δr and the detector resolution Δx

## Appendices

### Basics of muography

In this appendix we summarize the physical processes at the basis of the two main muography techniques. For more details, we recommend the introductory sections of^21^ and.^23^

#### Absorption-based muography

When passing through matter, muons lose energy through a variety of electromagnetic processes, among which the dominant one (up to energies of the order of hundreds of GeV) is ionization.^18^ Through macroscopic quantities of matter, the latter process can be approximated with a deterministic and continuous slowing down. If all the initial kinetic energy of a muon is dissipated while passing through an object, it will stop and then quickly decay. In absorption muography, a 2D opacity map *M* is extracted from the ratio of the flux through the object, as a function of the zenith (*θ*) and azimuth (*ϕ*) angles, over the corresponding flux in (θ,ϕ) when the detector is pointed toward an unobstructed direction (“free sky”). The opacity map is commonly extracted as:

(Equation 2) $$M\left(\theta ,\phi \right)=\frac{N_{obj}\left(\theta ,\phi \right)}{N_{fs}\left(\theta ,\phi \right)}\frac{\epsilon_{fs}\left(\theta ,\phi \right)}{\epsilon_{obj}\left(\theta ,\phi \right)}\frac{T_{fs}}{T_{obj}}\text{,}$$

where $N_{obj}\left(\theta ,\phi \right)$ and $N_{fs}\left(\theta ,\phi \right)$ indicate the number of muon tracks recorded as coming from the (θ,ϕ) direction when the detector points toward the object of interest (main run, or main dataset) or the free sky, respectively. The detector efficiency, indicated with $\epsilon_{obj/fs}\left(\theta ,\phi \right)$ here, is the probability that a real muon passes the selection criteria, which is in general non-uniform in (θ,ϕ) due to a variety of experimental nuisances such as failures of some electronic channels or miscalibration of their signals; obviously, $\epsilon_{obj}=\epsilon_{fs}$ when the same detector is used for the main run and the free-sky run, while this assumption cannot be made if different detectors are taking the main data and the free-sky ones simultaneously. Finally, $T_{obj}$ and $T_{fs}$ are the duration of the the main run and of the free-sky run, respectively, by which the muon counts need to be normalized in case they differ.

To be able to derive information about density, the measured M(θ,ϕ) must be compared with its expectation, which is a function of density *ρ*:

(Equation 3) $$M_{expected}\left(\theta ,\phi \right)=\frac{\int_{E_{\min}\left(X\right)}^{\infty }I\left(E;\theta ,\phi \right)dE}{\int_{E_{0}}^{\infty }I\left(E;\theta ,\phi \right)dE}\text{,}$$

where I(E;θ,ϕ) is the differential muon flux as function of energy (*E*) and direction (θ,phi). The value $E_{0}$ is the minimum energy for a muon to be detected (which depends on the specific tracking apparatus), while $E_{\min}$ is the minimum energy necessary for a muon to pass through material of opacity *X*. The latter is defined as the projection of the density (*ρ*) along the line of sight:

(Equation 4) $$X=\int_{entry}^{exit}\rho \;dx\text{,}$$

through the integration extreme in Equation 3:

(Equation 5) $$E_{\min}=E_{\min}^{rock}\left(X\right)+E_{0}\;\text{.}$$

Implicit in Equation 2 are a few simplifying assumptions.

(1) The direction (θ,ϕ) measured by the muography detector can be mapped unambiguously to trajectories within the objects, which amounts to neglecting both the intrinsic detector resolution and the deflection of muons through the matter of the object. A blurring of the image (irreducible, in the sense that it does not improve by simply accumulating more data) results from resolution and scattering effects.
(2) The distance between the object and the detector is such that the latter can be practically approximated as point-like. If this condition is not fulfilled, the information provided by (θ,ϕ) is not sufficient but must be complemented by the position of arrival of the muon on the face of the detector. On the other hand, in the backprojection algorithms (as discussed in STAR Methods and illustrated in Figure 22) this can be turned to our advantage to gain some degree of 3D information from a single muon telescope.
(3) The contamination from fake muons is negligible. Such contamination may originate from combinatorial background, i.e., tracks composed by random associations of hits that do not originate from a single particle, or from other charged particles such as electrons, positrons, protons, or pions, typically having lower energy than the muons. Methods to reduce these backgrounds are reviewed in a dedicated section of ref.^20^

The imaging resolution or resolving power Δr, defined as the precision in identifying a feature within the volume of interest, is obviously a function of the detector resolution Δx, which is defined as the precision at which we identify the position where a charged particle hits the detector. However, in all the applications of absorption muography discussed in state of the art and developments of interest in muography, the detector resolution is not the most crucial factor in determining the imaging resolution. The latter, indeed, depends on the angular resolution Δα and the distance *L* between detector and target (Δr≈LΔα), and Δα in turn depends on both the detector resolution Δx and the length l of the detector (distance between first and last plane) through Δx≈lΔα, as illustrated in Figure 23. Therefore, although small Δx always helps, by the interplay between l and *L* one often gets reasonable precision even with Δx of the order of centimeters, which is easy to achieve with any standard detection technique.

However, the ultimate limiting factor on the imaging resolution is the blurring due to the Coulomb scattering of the muons within the material of interest. This is among the crucial weaknesses of muography, together with the low muon flux at sea level.

#### Scattering-based muography

When traversing matter, the muons, like any other electrically charged particles, are deflected by Coulomb scattering due to the intense electric fields near nuclei. Single scattering, also called Rutherford scattering in honor of the scientist who studied the phenomenon more than a century ago, is described by the Rutherford formula^99^ that predicts some of the incoming particles to be deflected at very large angles. However, most deflections are small, so after traversing a macroscopic amount of material the number of scattering events is large and the combined effect of single scattering events (multiple scattering) can be approximated by Molière theory.^100^ Several parameterizations exist,^18^ where the scattering angle Δθ follows a Gaussian distribution for small angles and asymptotically converges to Rutherford formula at large angles. The standard deviation of the Gaussian core of the distribution tends to be well approximated by^101^:

(Equation 6) $$\sigma \left(\Delta \theta \right)=\frac{13.6\text{MeV}}{\beta cp}\sqrt{\frac{x}{X_{0}}}\left[1+0.038\;\ln\frac{x}{X_{0}\beta^{2}}\right]\text{,}$$

where *c* is the speed of light in vacuum, *β* is the relativistic velocity (defined as the ratio between the speed of the particle and *c*), *p* is the muon momentum, *x* is the path length through the object of interest, and the quantity $X_{0}$ is the macroscopic radiation length. It is homogeneous to a length [m] and is the ratio between the radiation length ($\Xi_{0}$) and the material’s density (*ρ*): $X_{0}\left[m\right]=\Xi_{0}\left[g.{\text{cm}}^{-2}\right]/\rho \left[g.{\text{cm}}^{-3}\right]$. The former is a characteristic of each material, function of the atomic number *Z* and of the atomic weight *A*, for which various approximations exist, for example^102^:

(Equation 7) $$\frac{1}{\Xi_{0}}\propto \frac{Z\left(Z+1\right)}{A}\ln\left(\frac{287}{\sqrt{Z}}\right)\;{\text{cm}}^{2}g^{-1}\;\text{.}$$

These formulas lead to the possibility to discriminate different materials by the scattering angle distributions or closely related observables.

### Momentum measurement

Both the absorption and the scattering methods, in their simplest implementation, can be applied without direct knowledge of the muon momentum. However, both can benefit from having access to the latter on a per-muon basis, even with low precision.^20^

While magnetic spectrometers are widespread in nuclear and particle physics research, they would be impractical or too expensive for most muography applications. Instead, some alternative muon momentum measurement methods have been investigated in the context of muography, which provide a rough measurement of momentum in exchange for a moderate increase in cost and complexity of the apparatus.

#### Scattering-based measurements

The relationship between multiple muon scattering and momentum, expressed in Equation 6, can be inverted to roughly infer momentum, provided that identity and amount of material are well known. This method works best for low-momentum muons. The tracking detector can be modified by introducing a thick wall of lead between the last and next-to-last panels of the tracker,^68^^,^^96^ or additional panels alternating with passive material.^103^^,^^104^ The proxy variable for momentum inference can be the scattering angle itself^68^ or closely related variables, such as the horizontal displacement with respect to the extrapolated trajectory or the goodness of fit ($\chi^{2}$) for the hypothesis of a straight track.^103^

#### Absorption-based filters

A simple momentum selection can be achieved by exploiting the stopping of muons by energy loss, i.e., the same physical mechanism at the basis of absorption-based muography. If a known thickness of passive material is positioned downstream of the tracking detector, it will stop all muons below a certain momentum, depending on its thickness times density. If an additional detector layer is installed after the passive material, the presence or absence of signal in that specific layer will indicate whether a muon (already selected through the other layers, upstream of the passive material) is above or below the momentum threshold, respectively. This can be employed to select high-momentum muons, to minimize the blurring effects due to the low-momentum ones,^96^ or to achieve the opposite goal of measuring low-momentum muons by using the lower panel as a veto.^82^ We advocate the latter in the simulated example of scenario II: average size regime.

#### Cherenkov radiation detectors

The Cherenkov effect is an electromagnetic phenomenon that is often exploited in particle physics experiments to measure the velocity of charged particles.^18^ In the context of muography, it can be used to determine whether a muon traversing a medium passes a momentum threshold (which depends on the refractive index of the medium) so that a sufficiently intense Cherenkov light signal is generated to be detected. A portable device that exploits this mechanism for measuring muon momentum with coarse resolution has been presented in ref.^105^ by using multiple pressurized gaseous Cherenkov radiators. The gas pressure at each radiator is selected to correspond to predefined muon momentum thresholds, so that a muon passing through the radiators only triggers those radiators with momentum threshold less than its actual momentum. By measuring the presence of Cherenkov signals in each radiator, it is then possible to classify individual muons in large (1 GeV resolution) bins of momentum with a true-positive rate of approximately 85% between 0.2 and 7.0 GeV.

### Statistical considerations for low-size objects

The specific energy loss of muons through ionization, dE/dx, normalized by the density *ρ* in order to get a quasi-material-independent number, is about $2\;MeVcm^{2}/g$ for a wide range of energies. For the typical stones used in monumental sculptures, *ρ* is in the ballpark of $2.5\;g/cm^{3}$ (e.g., $2.7\;g/cm^{3}$ for marble), meaning an energy loss of 5MeV/cm. Thus, a stone object with a thickness of 1 m absorbs the muons below ≈0.5GeV. With the help of the CRY parametric cosmic-ray simulation,^66^ we derived an approximate linear relationship between the energy cut-off, $E_{cut}$, and the fraction of muons above the cut-off (*f*) that is accurate at better than 1.5% level in the range between 100MeV and 2GeV: $f\approx \left(100-0.021E_{cut}\right)$%. From that, one sees that the fraction of muons <0.5GeV is only ≈10% of the total flux (with large uncertainties due to time- and location-dependent effects and various modeling issues), and even less if the detector is in a building, due to screening by its roof. So, while in typical applications of muography such as volcanoes or pyramids the size is such that only a tiny fraction of the *μ* flux survives the passage through the object of interest, for the use cases described in this article we are in the opposite regime. This may sound like an advantage of small objects, by enlarging the available statistics, but it is actually a limitation: our statistical power of discrimination depends on the fraction of muons that pass through the object (the same fraction *f* introduced earlier), and in the binomial approximation the statistical uncertainty is a function of f(1−f). Hence, both limits f→0 and f→1 lead to the same consequence: a vanishing statistical power.

Most muography studies are in the regime where f≪1 (e.g.,^47^), where the Poisson approximation is valid. In that regime, the relative statistical uncertainty scales as $1/\sqrt{N}$, where *N* is the number of passing muons. Here, instead, f≈1, hence the Poisson approximation fails. In our regime the binomial statistics is more appropriate, although still not exact: it is an approximation that holds only if *f* is not too close to 0 or 1. Close to those extreme values, the Clopper-Pearson approach^106^ is more accurate.
